# AttentionPert: accurately modeling multiplexed genetic perturbations with multi-scale effects

**DOI:** 10.1093/bioinformatics/btae244

**Published:** 2024-06-28

**Authors:** Ding Bai, Caleb N Ellington, Shentong Mo, Le Song, Eric P Xing

**Affiliations:** Machine Learning Department, Mohamed bin Zayed University of Artificial Intelligence, Abu Dhabi, 00000, United Arabic Emirates; Computational Biology Department, Carnegie Mellon University, Pittsburgh, PA, 15213, United States; Machine Learning Department, Mohamed bin Zayed University of Artificial Intelligence, Abu Dhabi, 00000, United Arabic Emirates; Machine Learning Department, Mohamed bin Zayed University of Artificial Intelligence, Abu Dhabi, 00000, United Arabic Emirates; Machine Learning Department, Mohamed bin Zayed University of Artificial Intelligence, Abu Dhabi, 00000, United Arabic Emirates; Machine Learning Department, Carnegie Mellon University, Pittsburgh, PA, 15213, United States

## Abstract

**Motivation:**

Genetic perturbations (e.g. knockouts, variants) have laid the foundation for our understanding of many diseases, implicating pathogenic mechanisms and indicating therapeutic targets. However, experimental assays are fundamentally limited by the number of measurable perturbations. Computational methods can fill this gap by predicting perturbation effects under novel conditions, but accurately predicting the transcriptional responses of cells to unseen perturbations remains a significant challenge.

**Results:**

We address this by developing a novel attention-based neural network, AttentionPert, which accurately predicts gene expression under multiplexed perturbations and generalizes to unseen conditions. AttentionPert integrates global and local effects in a multi-scale model, representing both the nonuniform system-wide impact of the genetic perturbation and the localized disturbance in a network of gene–gene similarities, enhancing its ability to predict nuanced transcriptional responses to both single and multi-gene perturbations. In comprehensive experiments, AttentionPert demonstrates superior performance across multiple datasets outperforming the state-of-the-art method in predicting differential gene expressions and revealing novel gene regulations. AttentionPert marks a significant improvement over current methods, particularly in handling the diversity of gene perturbations and in predicting out-of-distribution scenarios.

**Availability and implementation:**

Code is available at https://github.com/BaiDing1234/AttentionPert.

## 1 Introduction

Single-cell RNA-sequencing (scRNA-seq) has advanced significantly, enabling the generation of transcriptomic datasets encompassing millions of cell samples (Domcke *et al.* 2020, Han *et al.* 2020, The Tabula Muris Consortium 2020). This advancement facilitates CRISPR-based perturbational screens and rapid experimental sampling of genetic perturbation outcomes (Dixit *et al.* 2016, Norman *et al.* 2019, Frangieh *et al.* 2021, Przybyla and Gilbert 2022). However, this field faces significant challenges, particularly when expanding the number of genes for perturbation. The potential combinations of two-gene perturbations grow quadratically with an increasing number of genes (Norman *et al.* 2019), leading to a combinatorial explosion in scenarios involving even more simultaneous gene perturbations. Consequently, these methods are constrained by scalability and cost-effectiveness, especially when exploring new genetic perturbations (Dixit *et al.* 2016, Datlinger *et al.* 2017, Norman *et al.* 2019).

Many computational simulations model the gene regulatory networks (GRN), rather than directly incorporating the effects of perturbations, ranging from traditional Bayesian networks (Friedman *et al.* 2000) and causal inference algorithms (Wang *et al.* 2017) to more contemporary models such as SENIC (Aibar *et al.* 2017) and CellOracle (Kamimoto *et al.* 2023). Recent studies (Pratapa *et al.* 2020, Roohani *et al.* 2023) have indicated that achieving accurate GRN inference from transcriptomic data poses significant challenges, and GRN-predicting models often fall short in performance when tasked with predicting outcomes of single-gene and multi-gene perturbations. Moreover, other mechanistic modeling methods (Frohlich *et al.* 2018, Yuan *et al.* 2021) and linear models (Dixit *et al.* 2016, Kamimoto *et al.* 2023) addressing both chemical and genetic perturbations, face limitations including scalability issues in predicting responses for a vast number of genes, or an inability to capture nonlinear dynamics.

Current advancements in the field use deep neural networks trained on datasets encompassing extensive cells and genes with a moderate number of perturbations, which map genes, perturbations, or genetic relationships into a latent space for the prediction of perturbation outcomes (Lotfollahi *et al.* 2019 2023, Eraslan *et al.* 2019, Rampášek *et al.* 2019, Russkikh *et al.* 2020, Yu and Welch 2024, Roohani *et al.* 2023). Most of these methods, including scGEN (Lotfollahi *et al.* 2019), PerturbNet (Yu and Welch 2024), and CPA (Lotfollahi *et al.* 2023), primarily predict the transcriptional effects of chemical treatments rather than genetic perturbations. They often approach genetic perturbations as analogous to varying doses, which inadequately capture the complex interactions between different genes and the perturbed ones, resulting in limited performances in this particular domain (Lotfollahi *et al.* 2023).

Recently, there has been a growing trend in using large language models (LLMs) trained on unlabeled single-cell transcriptomic datasets, which often takes predicting perturbational effects as one of their downstream tasks (Cui *et al.* 2024, Gong *et al.* 2023, Hao *et al.* 2024, Yang *et al.* 2024). While these LLMs represent a relative advancement over other methodologies, they also benefit from downstream methods by incorporating expression representations generated by the foundational LLMs as inputs. Furthermore, the training of these foundation models requires extensive resources, utilizing large-scale datasets and numerous GPUs, which incurs significant time and financial costs.

Among all computational methods for genetic perturbation effects prediction, the state-of-the-art (SOTA) method graph-enhanced gene activation and repression simulator (GEARS) (Roohani *et al.* 2023), in contrast, specifically targets genetic perturbations. GEARS significantly surpasses other methods in this area, illustrating the effectiveness of a genetic-perturbation-centric preprocessing and training approach for future methodologies (Roohani *et al.* 2023). Current single-cell LLMs often take GEARS as a downstream probe in predicting perturbational effects (Cui *et al.* 2024, Gong *et al.*2023, Hao *et al.* 2024, Yang *et al.* 2024). A key innovation of GEARS is its incorporation of a highly expressive and informative knowledge graph based on Gene Ontology similarities. Nonetheless, GEARS as well as other methods still exhibit the three primary limitations that our work aims to address and advance.

We propose AttentionPert, a multi-level deep-learning method to accurately predict transcriptional responses to multiplexed genetic perturbations, which integrates the multi-head attention mechanism (Bahdanau *et al.* 2024) with graph neural networks on augmented gene interactions, alongside pre-trained co-expressive gene representations. AttentionPert is proposed to improve three major detriments of current deep-learning works on this problem. AttentionPert primarily improves existing methods by capturing nonuniform effects that the perturbation in a particular gene has on various other genes through the attention-based PertWeight encoder. Furthermore, AttentionPert accounts for localized gene perturbation analysis using the PertLocal encoder, complementing the global effects learned by PertWeight. PertLocal also avoids a pure linear combination of effects of perturbed genes and hence learns the nonadditive coeffects of multi-gene perturbations. Finally, AttentionPert utilizes a pre-trained context gene representation to initialize all the gene embeddings rather than random initialization.

AttentionPert outperforms prior methods including the SOTA GEARS model on *RPE1* and *K562* datasets (Replogle *et al.* 2022), each of which comprises >1000 single-gene perturbations, and on the *Norman* dataset containing 131 two-gene perturbations (Norman *et al.* 2019). Particularly, AttentionPert has exceptional capability in the most challenging out-of-distribution (OOD) task: predicting the effects of novel multi-gene perturbations where none of the perturbed genes have been encountered during the training phase. This remarkable performance highlights its ability to generalize effectively to entirely new genetic scenarios. Our experiments also reveal insights into nuanced genetic interactions associated with multi-gene perturbations through detailed gene-specific and perturbation-specific analyses.

## 2 Materials and methods

### 2.1 Problem setting

AttentionPert predicts post-perturbation gene expressions given specific gene-perturbation conditions. Formally, for a gene-perturbation condition denoted as $c=\{j_{1},j_{2},\ldots ,j_{m}\}\subseteq \{1,\ldots ,K\}$, representing a subset of gene indices, the objective is to predict the post-perturbation gene expression distribution $Y_{c}\in R^{K}$. Within the datasets, each perturbation is associated with multiple cells, of which post-perturbation expressions are represented as $y_{c}^{i}\in R^{K},i=1,\ldots ,n_{c}$, where *n_c_* is the number of cells corresponding to the condition *c*. A perturbation condition is termed *control* when *m *=* *0 and the unperturbed expressions are considered as known entities denoted by $\left\{x^{i},i=1,\ldots ,n_{\text{control}}\right\}$. The primary goal for a parametric model parameters $Y_{\theta }$ is to minimize the difference between the expected $Y_{\theta }(c)$ and the mean of the ground truth samples $\left\{y_{c}^{i}\right\}$ for each perturbation *c*.

Existing deep neural network models process both the perturbation condition and a randomly sampled unperturbed cell to predict a post-perturbation expression vector (Lotfollahi *et al.* 2023, Roohani *et al.* 2023). These predicted vectors are evaluated by measuring their similarity to actual perturbed samples. In our method, we adhere to the established preprocessing stage by randomly pairing each control vector $x_{c}^{i}$ from the set $\left\{x^{t},t=1,\ldots ,n_{\text{control}}\right\}$ with its corresponding post-perturbation vector $y_{c}^{i}$, where $i=1,\ldots ,n_{c}$ for each condition *c*. While CPA encodes gene expressions into a high-dimensional space and then perturbs them within this space (Lotfollahi *et al.* 2023), our approach follows GEARS, focusing on directly predicting the expression shift resulting from the perturbation condition and adding it to the control expression, formally expressed as: ${\hat{y}}_{\theta }{\left(c\right)}^{i}=g_{\theta }(c)\;+\;x_{c}^{i}$. We also adopt the combined loss function used by GEARS, which integrates auto-focus and direction-aware loss, to compare our predictions ${\hat{y}}_{\theta }{\left(c\right)}^{i}$ with the ground-truth $y_{c}^{i}$. We detail the preprocessing and loss function formulas in Supplementary Section S1.1. The primary innovation of our model is the improved post-perturbation expression change predictor $g_{\theta }$.

### 2.2 Model overview

**Figure 1.**
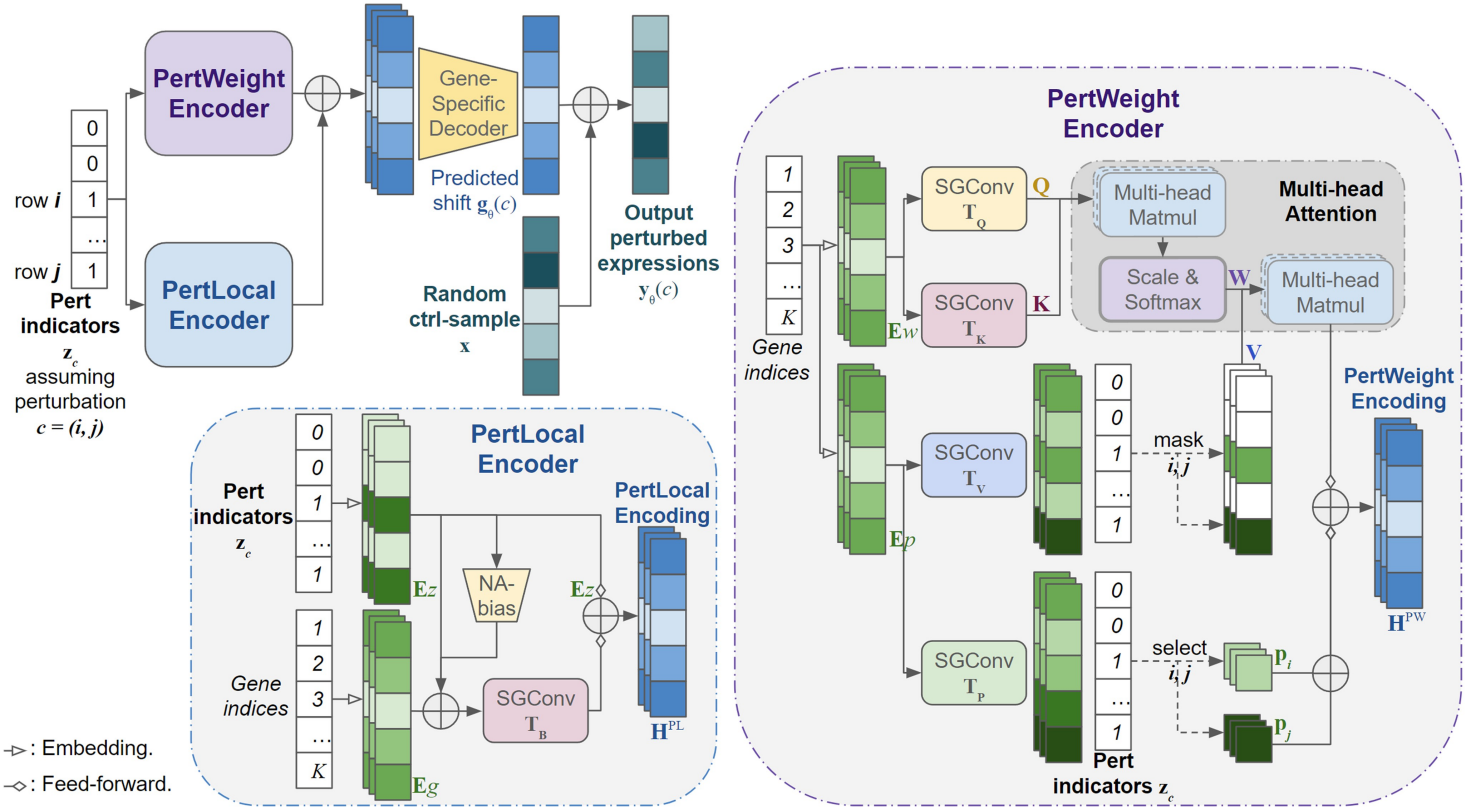
The overall architecture of AttentionPert. The illustration assumes that the perturbation condition c={i,j}. The indicators vector $z_{c}$ is the input of both the PertWeight Encoder and the PertLocal Encoder. PertWeight generates attention weight matrices **W** based on the embedding $E_{w}$, uses the perturbation indicators to filter the values **V** and **P**, and then combines both the weighted and the general global effects. PertLocal takes the perturbation indicators and outputs the locally disturbed gene states. The gene-specific linear decoder decodes the sum of two encodings into the perturbation effects, and then the predicted expressions are obtained. All SGConvs of PertWeight: $T_{Q},T_{K},T_{V}$ and $T_{P}$ take the Gene Ontology graph as the input graph, while the SGConv of PertLocal $T_{B}$ takes the augmented GO graph. All embeddings for gene indexes are initialized using pre-trained Gene2Vec embeddings and can be learned during training. All the feed-forward layers are multiple linear and batch normalization layers.

AttentionPert takes a perturbation condition c⊆{1,…,K} as input. The vector $z_{c}\in {\left\{0,1\right\}}^{K}$ contains the 0/1 perturbation indicators, formally: $z_{c,j}=1$ if j∈c and 0 otherwise. AttentionPert feeds the perturbation indicators into two modules called PertWeight and PertLocal encoders respectively. Each encoder outputs a matrix of encoding, noted as $H^{\text{PW}}$ by PertWeight and $H^{\text{PL}}$ by PertLocal, which both $\in R^{K\;\times \;D}$, where *D* is the network hidden size. After that, a gene-specific decoder takes the sum of two encodings and then generates the predicted post-perturbation expression shift $g_{\theta }(c)$, as shown in Fig. 1. Finally, we add the shift to a random control sample $x_{c}^{i}$ to get a post-perturbation prediction ${\hat{y}}_{\theta }{\left(c\right)}^{i}=g_{\theta }(c)\;+\;x_{c}^{i}$, and compute the loss function for the whole batch to learn the model parameter *θ*. A more intuitive illustration of this method is Supplementary Section S1.2.

### 2.3 PertWeight for nonuniform global effects

Current works treat all affected genes uniformly when computing the effects of a genetic perturbation in the embedding space, which does not differentiate how a perturbed gene might impact other genes in unique ways (Lotfollahi *et al.* 2019, 2023, Roohani *et al.* 2023). To address this issue, the attention-based perturbation encoder PertWeight is designed to learn weight matrices representing the interactions from perturbed genes to other genes. These matrices are then applied in a weighted addition to compute post-perturbation states, thus enabling the model to capture the nonuniform gene-to-gene perturbational effects.

PertWeight firstly embeds gene indices {1,2,…K} into two independent sets of embeddings. We define the first embedding as the *weight* embedding noted by $E_{w}$, and the second embedding as the *pert* embedding noted by $E_{p}$. Here: $E_{w},E_{p}\in R^{K\;\times \;D_{e}}$. Both embeddings can be either randomly initialized or loaded with the pre-trained gene-representing embeddings. Current studies assign each gene a high-dimensional representation that is randomly initialized (Lotfollahi *et al.* 2023, Roohani *et al.* 2023), which is a suboptimal approach considering the limited range of experimental perturbation conditions available in comparison to the potential perturbations. Utilizing pre-trained context representations for initializing gene embeddings could significantly enhance the model’s ability to predict responses to unseen perturbed genes. Our proposed method incorporates Gene2Vec (Du *et al.* 2019), which offers 200D vector representations for human genes, derived from gene co-expression patterns across 984 datasets in the Gene Expression Omnibus (GEO) databases. It is proven to be more expressive through our experiments than random initializing gene representations.

In this module, we apply four convolutional graph neural network (GNN) encoders with the Gene Ontology (GO) graph as the input graph J with an adjacency matrix **J**. The GNN model *SGConv* (Li *et al.* 2024) has linear projection weights $T\in R^{D_{\text{in}}\;\times \;D_{\text{out}}}$. Taking the input graph node embedding $E\in R^{K\;\times \;D_{\text{in}}}$ and the graph A of *K* nodes with an adjacency matrix $A\in R^{K\;\times \;K}$, a *t*-hop SGConv outputs a graph-convolution encoding ${\text{SGC}}_{t,T}(A,E)\in R^{K\;\times \;D_{\text{out}}}$.

A Gene Ontology term provides a structured framework to represent information in a specific domain, where every gene is associated with multiple Gene Ontology terms (Carbon *et al.* 2009, Thomas *et al.* 2019, 2022). The GO graph J is constructed through Gene Ontology following the SOTA method GEARS (Roohani *et al.* 2023), where the edge weight of two genes is the fraction of their shared Gene Ontology terms. Details for the GO graph are shown in Supplementary Section S1.3.

The four SGConv encoders have different weight matrices: $T_{Q}\in R^{D_{e}\;\times \;Hd_{q}},T_{K}\in R^{D_{e}\;\times \;Hd_{k}},T_{V}\in R^{D_{e}\;\times \;Hd_{v}}$ and $T_{P}\in R^{D_{e}\;\times \;D_{p}}$ respectively, where *H* is the number of heads in the multi-head attention, and *d_q_* = *d_k_*. SGConvs $T_{Q}$ and $T_{K}$ process the weight embedding $E_{w}$ and SGConvs $T_{V}$ and $T_{P}$ process the pert embedding $E_{p}$. Then we got four outputs:

(1) $$\{\begin{array}{cc} Q & ={\text{SGC}}_{t,T_{Q}}(J,E_{w})\in R^{K\;\times \;Hd_{q}}\\ K & ={\text{SGC}}_{t,T_{K}}(J,E_{w})\in R^{K\;\times \;Hd_{k}}\\ V & ={\text{SGC}}_{t,T_{V}}(J,E_{p})\cdot z_{c}\in R^{K\;\times \;Hd_{v}}\\ P & ={\text{SGC}}_{t,T_{P}}(J,E_{p})\in R^{K\;\times \;D_{p}} \end{array}$$

Note that here the output of the SGConv $T_{V}$ is masked using the vector of perturbation indicators $z_{c}$, leaving only the values of perturbed genes. **Q**, **K** and **V** are then fed into the multi-head attention as queries, keys and values (Bahdanau *et al.* 2024), as shown in Fig. 1. Specifically, queries, keys and values are all equally divided into *H* matrices along the second dimension. Note that for head *h*, the sub-matrices are $Q^{h},\;K^{h}$ and $V^{h}$, and the attention matrix of each head is:

(2) $$W^{h}=\text{softmax}(\frac{Q^{h}{\left(K^{h}\right)}^{T}}{\sqrt{d_{k}}})$$

And the output of the multi-head attention $H^{\text{MHA}}$ is the concatenation of all heads $H^{\text{MHA},h}=W^{h}V^{h}$. Since **V** is masked by the vector of perturbation indicators $z_{c}$, the output can also be written as:

(3) $$h_{k}^{\text{MHA},h}=\sum_{j\in c}W_{kj}^{h}v_{j}^{h}$$

for each gene k∈{1,…,K}. This is a weighted summation of perturbational effects from all perturbed genes. All attention matrices $W^{h}\in R^{K\;\times \;K}$ are the *Perturbation Weight Matrices*, which are the key innovation of PertWeight module featuring the various effects between any perturbed genes and all genes.

The output **P** represents the general effects of perturbed genes upon all genes. The row vectors of **P** in the perturbed genes $\left\{p_{j}|j\in c\right\}$ are selected and added to the final output. Hence the final output of the PertWeight Encoder is:

(4) $$h_{k}^{\text{PW}}=f_{1}(h_{k}^{\text{MHA}})\;+\;f_{2}(\sum_{j\in c}p_{j})$$

for each gene *k*, where $f_{1}$ and $f_{2}$ are linear feed-forward layers that project encodings into the same hidden size *D*, followed by layer-normalization. The output matrix is $H^{\text{PW}}={\left[h_{1}^{\text{PW}},\ldots ,h_{K}^{\text{PW}}\right]}^{T}\in R^{K\;\times \;D}$.

Obviously, PertWeight is additive for genes in any multi-gene perturbation *c*, which cannot simulate the nonadditive feature, of which details are shown in Supplementary Section S1.4. Therefore, we propose PertLocal which learns the nonlinear co-effects of perturbations of multiple genes.

### 2.4 PertLocal for local disturbance

Many existing models primarily focus on the perturbational effects across all genes within a cell (Lotfollahi *et al.* 2019, 2023), overlooking the different interactions between genes. The SOTA method uses a gene–gene interaction graph, yet it only models global effects (Roohani *et al.* 2023). However, genetic regulation studies demonstrate that local and global perturbational effects complement each other. PertLocal is designed to overcome this gap by learning the localized disturbance effects of perturbed genes on their closely related counterparts. It complements the global effects captured by the PertWeight encoder, thereby significantly enhancing the overall predictive accuracy of the model.

Similar to PertWeight, PertLocal also starts with two embeddings. The first one is the gene base embedding representing unperturbed states of genes that embeds gene indices {1,2,…K} into dimension *D_e_*, and the second one is the perturbation indicator embedding that embeds binary indicators {0, 1} into dimension *D_e_*. The indicator embedding layer embeds the perturbation indicator vector $z_{c}\in {\left\{0,1\right\}}^{K}$ into a matrix of dimension $K\;\times \;D_{e}$. We denote the gene base embedding as $E_{g}\in R^{K\;\times \;D_{e}}$ and the perturbation indicator embedding as $E_{z}\in R^{K\;\times \;D_{e}}$. Similarly to $E_{w}$ and $E_{p}$, the gene base embedding $E_{g}$ is also initialized with Gene2Vec.

**Figure 2.**
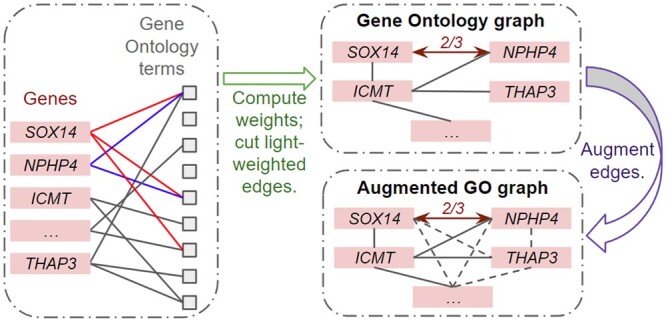
The process to obtain the Gene Ontology (GO) graph and the augmented GO graph. Using the GO terms of each pair of genes, e.g. those marked in red for gene SOX14 and those marked in blue for NPHP4, the edge weight is defined as the number of shared GO terms divided by the size of the union set of their GO terms, which is 2/3 for genes SOX14 and NPHP4 (only as an illustration in the figure). The GO graph is then obtained by filtering 20 highest edge weights for each gene and cutting all other edges. The augmented GO graph is fully connected, in which each edge either exists in the GO graph or is augmented as *α* times the minimum edge weight of the GO graph, where α∈(0,1].

Numerous previous studies have inaccurately used the additive prediction approach commonly used in multi-gene perturbation modeling (Friedman *et al.* 2000, Aibar *et al.* 2017, Wang *et al.* 2017, Kamimoto *et al.* 2023), which the SOTA work designed an innovative cross-gene layer to avoid (Roohani *et al.* 2023). In contrast, PertLocal adopts a nonadditive bias layer to effectively learn the complex, nonlinear coeffect resulting from multiple perturbed genes. The nonadditive (NA) biased embedding for the perturbation indicator embedding is formally:

(5) $${E\prime }_{z}=E_{z}[1\;+\;\beta (m\;-\;1)\tanh(K_{\text{NA}}z_{c})]$$

where β∈(0,1) is a hyper-parameter, m=|c| is the number of perturbed genes, and $K_{\text{NA}}\in R^{D_{e}\;\times \;K}$ is a trainable weight matrix. PertLocal then applies another *t*-hop SGConv encoder on the summation of the two embeddings $E_{g}\;+\;E_{z}^{\prime }$, which takes the augmented Gene Ontology graph as the input graph. The augmented GO graph J′ is created by adding virtual edges to all disconnected pairs of nodes in the GO graph with a new minimum edge weight. The process to attain the GO graph and the augmented GO graph is shown in Fig. 2. Formally,

(6) $${J\prime }_{ij}=\{\begin{array}{c} J_{ij},J_{ij}\;>\;0\\ \alpha \min_{k,l}\{J_{kl}|J_{kl}\;>\;0\},J_{ij}=0 \end{array}$$

where 0 < α < =1. Virtually added edges with minimum weights make the graph J′ fully connected, which allows PertLocal to learn lightweight instead of zero global gene–gene reactions. The details of the GO graph and the GNN encoders can be found in Supplementary Section S1.3.

Denote the weight matrix for the PertLocal SGConv as $T_{B}\in R^{D_{e}\;\times \;D_{b}}$. After two feed-forward and then batch normalization layers $f_{3}$ and $f_{4}$ of the same output size *D*, the SGConv output and the perturbation indicator embedding are then added together as the output of the PertLocal Encoder:

(7) $$H^{\text{PL}}=f_{3}({\text{SGC}}_{t,T_{B}}(J\prime ,E_{g}\;+\;{E\prime }_{z}))\;+\;f_{4}(E_{z})$$

as shown in Fig. 1.

The PertLocal encoder is nonadditive since the NA-biased embedding is identical to the indicator embedding when *m *=* *1 but not when m ≥ 2, which then induces the nonadditive property (Supplementary Section S1.4). Through the weighted gene–gene graph structure and the indicator embedding, PertLocal learns the representations of local perturbational effects that complement the PertWeight encoding.

### 2.5 Decoder for transcriptional effects

After getting the encodings from both encoders, we input the sum of $H^{\text{PW}}$ and $H^{\text{PL}}$ into a linear decoder, which simplifies GEARS decoder by removing its cross-gene part. In this decoder, after a feed-forward function for each gene *k*, a gene-specific linear layer maps the post-perturbation encoding into a scalar that is the predicted perturbation effect. Formally:

(8) $$g_{\theta }{\left(c\right)}_{k}=w_{k}^{T}f_{5}(h_{k}^{\text{PW}}\;+\;h_{k}^{\text{PL}})\;+\;b_{k}$$

where $w_{k}\in R^{D}$ and $b_{k}\in R$ are learnable weights and bias of the linear projection for each gene. The cross-gene layer is used in GEARS for predicting the nonadditive effects of multi-gene perturbations, which is covered in our PertLocal model. Since PertWeight and PertLocal have learned both the uneven global and the local perturbational effects, a relatively less expressive cross-gene layer is abandoned to avoid redundancy. Experiment results demonstrate that AttentionPert achieves superior results, outperforming the need for a cross-gene layer.

The final output is the predicted post-perturbation gene expressions ${\hat{y}}_{\theta }{\left(c\right)}^{i}$ obtained by adding the predicted post-perturbation expression shift vector $g_{\theta }(c)$ to a randomly sampled unperturbed control gene expressions $x_{c}^{i}$, as shown in Fig. 1. The combined loss function (inherited from GEARS) is computed by taking the outputs of a mini-batch and then optimized to learn the parameters of $g_{\theta }$.

## 3 Results

### 3.1 Experimental setup

**Evaluation metrics.** The evaluation metrics for each perturbation condition *c* is the mean squared error (MSE) between the mean prediction and the average ground-truth post-perturbation expression on the top 20 differentially expressed (DE) genes. We also evaluate the Pearson correlation coefficient between the predicted post-perturbation expression shift and the ground-truth shift $\rho_{\Delta }$, which is evaluated throughout all genes to test the overall performance. Detailed descriptions of metrics are shown in Supplementary Section S2. Test results on other numbers of top DE genes are shown in Supplementary Section S5.3.

**Baselines.** We conduct experiments for three baselines and compare their performances with AttentionPert. The first one is the model *Ctrl* that directly outputs the mean value of all unperturbed control expressions, which is hence the worst case for any dataset. The performance of *Ctrl* also indicates the difficulty of the post-perturbation prediction since it measures how much effect those perturbations have on unperturbed cells. Due to the differences between datasets and perturbations inside the same dataset, the difficulty of predicting the effect of novel gene perturbations varies much. Hence, we also calculate the relative mean squared error (*rel-MSE*) for each model that is its MSE divided by the MSE of *Ctrl*. The other two baselines are CPA (Lotfollahi *et al.* 2023) and GEARS (Roohani *et al.* 2023).

**Implementation and training.** AttentionPert is implemented with the embedding size *D_e_* = 200 of all embeddings fit the Gene2Vec. All hyper-parameters of AttentionPert are set as (Supplementary Section S3): the hop number of all SGConvs *t *=* *1; the output dimension of SGConvs $T_{P}$ and $T_{B}$ equals to the network hidden size $D_{p}=D_{B}=D=64$; the number of heads *H *=* *2 and the head dimension $d_{q}=d_{k}=d_{v}=64$ in the multi-head attention of the PertWeight encoder; the minimum edge weight of the GO graph is multiplied by α=0.75 when generating the Augmented GO graph, and the NA-bias parameter β=0.05. We train the model in each data split independently for 20 epochs, with a batch size 128 and an Adam optimizer with a learning rate $10^{\;-\;3}$. We run the training process for all models on five splits of three datasets that are Norman, RPE1 and K562 (Norman *et al.* 2019, Replogle *et al.* 2022) preprocessed by GEARS (Roohani *et al.* 2023), of which details are shown in Supplementary Section S4. Except for *Ctrl* which is nonparameterized, we conduct each experiment five times with randomly different model initialization and take the average and standard deviation of the results.

### 3.2 Comparison to prior work

The high cost of CRISPR gene-perturbation screens requires an in-silico method that predicts transcriptomic effects of novel single/multi-gene perturbations not in the existing datasets. Therefore in our experiments, all the cells of a given perturbation condition *c* are either in the train and validation set or in the test set, which simulates the task of predicting unseen perturbations. For single-gene perturbations, we define only one generalization class called *seen 0/1* where the only perturbed gene is never seen in the train and validation set. When considering the combination of multiple perturbed genes, however, there are multiple testing scenarios, depending on the experimental exposure of the genes to perturbations during training.

AttentionPert is adept at predicting transcriptional outcomes for perturbations involving multiple genes, of which performance is evaluated using the Norman dataset (Norman *et al.* 2019) that comprised 131 two-gene perturbations. As previously mentioned, two-gene perturbations in the test set are divided into three generalization classes for evaluation purposes. The first class includes scenarios where both genes in a two-gene perturbation set are individually encountered in the training data (*2-gene perturbation, seen 2/2*). The more challenging scenarios involve cases where either one gene (*seen 1/2*) or neither gene (*seen 0/2*) of the two-gene set are perturbed in the training dataset. The comparative analysis of these three scenarios is detailed in Table 1. The results demonstrate that AttentionPert outperforms all baseline models across these scenarios, particularly in the most OOD scenario where both test genes are unseen in the train set.

**Table 1.** Seen 0/2, seen 1/2, and seen 2/2 comparison on Split 1 of Norman dataset.^a^

Model	Seen 0/2			Seen 1/2			Seen 2/2		
	MSE(DE)	rel-MSE(DE)	$\rho_{\Delta }$ (all)	MSE(DE)	rel-MSE(DE)	$\rho_{\Delta }$ (all)	MSE(DE)	rel-MSE(DE)	$\rho_{\Delta }$ (all)
Ctrl	0.503	100	0.013	0.689	100	0.016	0.604	100	–0.016
CPA	0.243 ± 0.032	48.3 ± 6.3	0.517 ± 0.026	0.388 ± 0.010	56.3 ± 1.4	0.577 ± 0.005	0.321 ± 0.026	53.2 ± 4.3	0.573 ± 0.005
GEARS	0.219 ± 0.011	43.5 ± 2.2	0.558 ± 0.016	0.221 ± 0.007	32.1 ± 0.9	0.595 ± 0.015	0.130 ± 0.007	21.6 ± 1.1	0.657 ± 0.027
Ours	**0.159** ± 0.012	**31.6** ± 2.5	**0.574** ± 0.016	**0.193** ± 0.003	**28.0** ± 0.4	**0.617** ± 0.005	**0.097** ± 0.008	**16.1** ± 1.4	**0.684** ± 0.013

a MSE(DE) is the MSE of the top 20 DE genes. The relative MSE(DE) is in percentage (%) and is relative to the Ctrl model. The ρΔ score refers to the Pearson score between the predicted shift and the ground-truth shift. We record the mean and the standard deviation over five independent experiments. The best performances are marked in bold.

**Figure 3.**
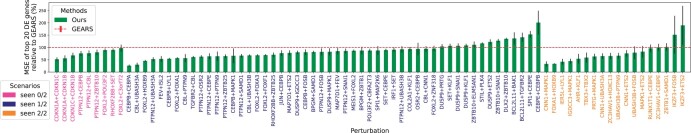
Perturbation-specific comparison on Split 1 of Norman dataset. The MSE(DE) values of AttentionPert are normalized as percentages relative to the performance of GEARS.

Furthermore, Fig. 3 shows the specific comparison between AttentionPert and the SOTA method GEARS for 79 tested two-gene perturbations of Split 1 of Norman dataset. Among them, AttentionPert outperforms GEARS on all nine perturbations of the hardest seen 0/2 scenario. Over three scenarios, AttentionPert has better performances on 63 perturbations, which is about 80% of the test cases. This reveals that AttentionPert surpasses the SOTA method in predicting the effects of two-gene perturbations.

**Figure 4.**
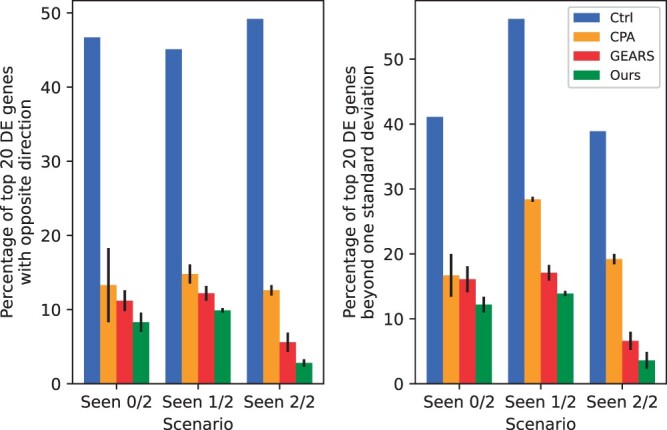
Comparison of metrics representing error rates on Split 1 of Norman dataset. These two metrics evaluate the average proportion of genes with incorrect direction and value predictions, respectively.

We apply two metrics evaluating the average error rate when dealing with different genes apart from MSE(DE) and $\rho_{\Delta }$(all) which represent overall performances across the set of genes. The first metric quantifies the proportion of the top 20 DE genes for a given perturbation predicted to change in an opposite direction compared to their actual change. The second metric is the percentage of DE genes for which the predicted post-perturbation gene expression falls outside one standard deviation of the actual post-perturbation expression distribution mean. Both metrics describe the ratio of wrongly predicted genes, of which lower values are preferable. Figure 4 shows the comparison between baseline models and AttentionPert on these two scores, demonstrating that our model makes fewer errors in predicting both the direction and value of perturbation effects for the top 20 DE genes.

For single-gene perturbations, we used datasets from two separate genetic perturbation screens: one involving RPE1 cells with 1543 perturbations and another with K562 cells, comprising 1092 perturbations. Each dataset contained data from over 160 000 cells (Replogle *et al.* 2022). The performance of AttentionPert is compared against baseline models, as detailed in Table 2. The results demonstrate that AttentionPert outperforms all baselines in all metrics.

**Table 2.** Seen 0/1 comparison on Split 1 of RPE1 and K562 datasets.

Data	Model	MSE(DE)	rel-MSE(DE)	$\rho_{\Delta }$ **(all)**
RPE1	Ctrl	0.290	100	–0.017
	CPA	0.281 ± 0.001	97.0 ± 0.4	0.109 ± 0.025
	GEARS	0.143 ± 0.005	49.3 ± 1.6	0.527 ± 0.025
	Ours	**0.132** ± 0.005	**45.7** ± 1.7	**0.532** ± 0.010
K562	Ctrl	0.211	100	–0.014
	CPA	0.205 ± 0.000	97.3 ± 0.1	0.088 ± 0.015
	GEARS	0.137 ± 0.003	65.1 ± 1.2	0.327 ± 0.015
	Ours	**0.132** ± 0.003	**62.4** ± 1.3	**0.340** ± 0.012

The best performances are marked in bold.

Results in Supplementary Section S5.1 show that AttentionPert beats all the baselines on all other four splits of each dataset, which underscores the robustness and adaptability of AttentionPert under diverse test conditions.

### 3.3 Discovering genetic interactions

**Figure 5.**
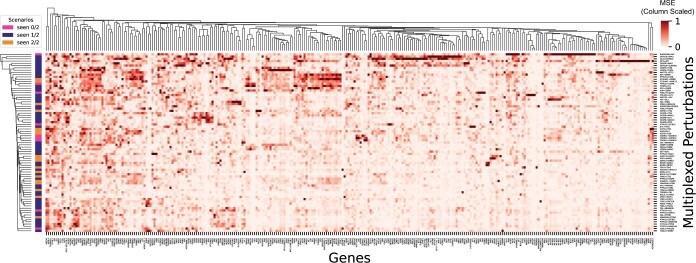
Column-scaled MSEs of AttentionPert. The results are shown over 79 tested 2-gene perturbations of Split 1 in the Norman dataset as rows and the union set of the top 20 DE genes of these perturbations as columns.

Non-Gaussianity in residual errors reveals outlier complexity in data not captured by the model. Here, we analyze AttentionPert’s residual errors to diagnose any failure cases in the model, while also revealing nuanced genetic interactions, outlier complexity in the regulation of some genes, and gene modules with correlated errors (Fig. 5). AttentionPert achieves near-zero error on perturbations to the FOX* transcription factor family across all genes, suggesting that these are degenerate cases where the complexity of the transcriptome decreases in their absence. In contrast, perturbation cases with ZBTB10, SNAI1, or SET have much higher residuals than average, possibly indicating an induced incoherent system or cellular decay due to their broad cellular functions.

Notably, the residuals are uncorrelated with the perturbation domain splits (Fig. 5), and uncorrelated with the structure of the Gene Ontology graph (Supplementary Fig. S9). Complementing AttentionPert’s SOTA performance on all domain generalization tasks (Table 1), this gives us high confidence that AttentionPert generalizes extremely well, and that high error genes and perturbations are indicative of complex and nuanced genetic mechanisms. More analysis of perturbation-gene residual errors is in Supplementary Section S6.

### 3.4 Predicting nonadditive perturbation effects

**Figure 6.**
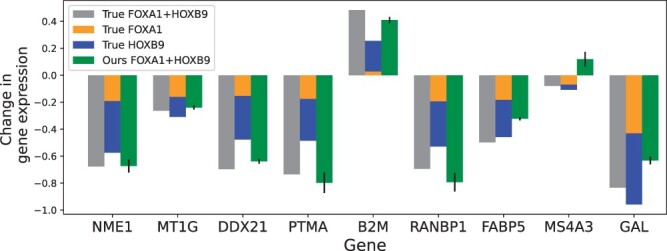
Change in gene expression after perturbing the combination FOXA1 and HOXB9.The true mean post-perturbation gene expression change, the sum of true changes for the two individual single-gene perturbations, and the change predicted by AttentionPert for each gene out of 9 example genes.

Nonadditive effects arising from the complex interactions between multiple perturbed genes underscore the necessity for a model that can accurately and integrally handle multi-gene perturbations. Figure 6 illustrates the ability of AttentionPert to capture the nonadditive nature of multiplexed genetic perturbations. When predicting a seen 2/2 perturbation: FOXA1 and HOXB9, AttentionPert is adept at learning the combined response of these genes. This approach differs markedly from a simple additive model, which merely sums the transcriptional effects of each gene perturbed independently.

Furthermore, Table 3 compares AttentionPert with or without NA-bias on nonadditive genes (NAG). NAG is the subset of the top 200 DE genes given a 2-gene perturbation that expresses nonadditivity above the threshold. Formally, given a 2-gene perturbation c={i,j} and a threshold *η*, ${\text{NAG}}_{c}=\{k|k\in \text{top}\;200\;\text{DE genes},|\Delta_{\{i\},k}\;+\;\Delta_{\{j\},k}-\Delta_{c,k}|\;\geq \;\eta |\Delta_{c,k}|\}$, where $\Delta_{c,k}$ is the true expression effect of perturbation *c* on gene *k*. Results demonstrate that AttentionPert with NA-bias more accurately predicts nonadditive expression changes. Here, the number 200 rather than 20 is used to select more NAGs. For η=0.25,0.5,0.75, the average proportion of NAGs out of 200 DE genes is 66.3%,40.3%,22.6% respectively.

**Table 3.** Comparison on Split 1 of Norman dataset for different thresholds of nonadditive genes.^a^

*η*	NA-bias	Seen 0/2	Seen 1/2	Seen 2/2
		MSE(NAG)	MSE(NAG)	MSE(NAG)
0.25	no	0.0374 ± 0.0024	0.0424 ± 0.0020	0.0354 ± 0.0016
	yes	**0.0331** ± 0.0022	**0.0406** ± 0.0007	**0.0331** ± 0.0030
0.5	no	0.0298 ± 0.0036	0.0320 ± 0.0031	0.0374 ± 0.0026
	yes	**0.0258** ± 0.0018	**0.0301** ± 0.0009	**0.0354** ± 0.0039
0.75	no	0.0275 ± 0.0043	0.0234 ± 0.0034	0.0379 ± 0.0043
	yes	**0.0225** ± 0.0020	**0.0218** ± 0.0011	**0.0371** ± 0.0048

a Here nonadditive genes (*NAG*) over a certain threshold *η* is a subset {*k*} of top 200 DE genes given a 2-gene perturbation c={i,j} where $\left|\Delta_{\{i\},k}+\Delta_{\{j\},k}-\Delta_{c,k}|\geq \eta |\Delta_{c,k}\right|$ (the nonadditive effect is larger than *η* times the true expression change). The best performances are marked in bold.

### 3.5 Ablation study

The primary distinction between AttentionPert and the SOTA GEARS lies in the expression shift model architecture, $g_{\theta }$. Besides, AttentionPert differs from GEARS in three aspects: first, we discard the cross-gene layer utilized by GEARS; second, we match the number of potentially perturbed genes to the size of the genes in the dataset, while GEARS includes a different set of potential perturbed genes, adding numerous nodes to the GO graph that are not perturbed in actual datasets; third, we utilize pre-trained Gene2Vec embeddings for initializing all gene embeddings in AttentionPert, while GEARS uses random initialization. The ablation study presented in Table 4 demonstrates that AttentionPert achieves the lowest MSE(DE) in the seen 0/2 scenario, which indicates that the enhanced performance of AttentionPert is not solely attributable to any one of these three changes or their combination. Therefore, the design of AttentionPert is essential for its improved performance.

**Table 4.** Ablation experimental results comparing other differences from GEARS.^a^

Model	CG	Rd	G2V	Seen 0/2	Seen 1/2	Seen 2/2
				MSE(DE)	MSE(DE)	MSE(DE)
GEARS	✓			0.219 ± 0.011	0.221 ± 0.007	0.130 ± 0.007
GEARS	✓		✓	0.293 ± 0.010	0.449 ± 0.009	0.391 ± 0.006
GEARS	✓	✓		0.235 ± 0.006	0.234 ± 0.008	0.117 ± 0.004
GEARS	✓	✓	✓	0.194 ± 0.012	0.225 ± 0.007	0.110 ± 0.003
GEARS		✓		0.246 ± 0.022	0.215 ± 0.011	**0.087** ± 0.003
GEARS		✓	✓	0.208 ± 0.015	0.211 ± 0.005	0.094 ± 0.004
Ours		✓	✓	**0.159** ± 0.012	**0.193** ± 0.003	0.097 ± 0.008

a *CG* means utilizing the cross-gene decoding layer. *Rd* means reducing the potentially perturbed genes. *G2V* is initializing the gene embeddings with Gene2Vec. The original GEARS method utilizes cross-gene without reducing genes or using Gene2Vec. The best performances are marked in bold.

In our study, we conduct ablation experiments on the two encoders: PertWeight and PertLocal. The results presented in Table 5 reveal that while each encoder contributes to the model’s performance, neither encoder individually outperforms the effectiveness of the integrated model that combines both encoders. This underscores the synergistic effect of incorporating both the PertWeight and PertLocal encoders into AttentionPert. Besides, results in Tables 4 and 5 show that AttentionPert without Gene2Vec beats revised versions of GEARS but has worse results than the final version of AttentionPert where we use Gene2Vec to initialize gene embeddings. Comparisons of AttentionPert with or without the NA-bias module shown in Tables 3 and 5 demonstrate that NA-bias inside PertLocal enhances the performance of AttentionPert.

**Table 5.** Ablation experimental results on AttentionPert.^a^

Model	NA	PL	PW	G2V	Seen 0/2	Seen 1/2	Seen 2/2
					MSE(DE)	MSE(DE)	MSE(DE)
PL	✓	✓		✓	0.246 ± 0.002	0.388 ± 0.004	0.324 ± 0.002
PW			✓	✓	0.172 ± 0.009	0.202 ± 0.004	0.120 ± 0.007
PL+PW		✓	✓	✓	0.168 ± 0.009	0.196 ± 0.004	0.104 ± 0.010
PL+PW	✓	✓	✓		0.189 ± 0.026	0.203 ± 0.009	0.105 ± 0.018
Ours	✓	✓	✓	✓	**0.159** ± 0.012	**0.193** ± 0.003	**0.097** ± 0.008

a We compare our model with/without each of the four components: nonadditive bias (*NA*), PertLocal (*PL*), PertWeight (*PW*), and Gene2Vec (*G2V*) initialization. NA is an optional design only inside the PL module. The best performances are marked in bold.

Ablation study results for other splits are detailed in Supplementary Section S5.2. The ablation study conclusively demonstrates that each component of AttentionPert contributes to its overall performance.

## 4 Conclusion

We have proposed a comprehensive model AttentionPert that effectively integrates attention mechanisms to predict the unique effects of single or multiple perturbed genes. By incorporating the PertWeight and PertLocal encoders that complement each other with encodings representing global and local perturbational effects respectively, we have successfully addressed the challenges of predicting transcriptional responses to genetic perturbations. AttentionPert not only outperforms existing state-of-the-art methods in terms of accuracy and handling diverse gene perturbations but also shows exceptional capability in predicting out-of-distribution scenarios where all simultaneously perturbed genes are unseen. The demonstrated superior performance across multiple datasets highlights its potential in facilitating medical research and treatment development. Besides, detailed perturbation-gene-specific experimental analyses on residual errors provide valuable insights into the intricate genetic interactions under various perturbation conditions. Furthermore, by using the innovative NA-bias module, AttentionPert predicts accurate nonadditive effects of multiplexed perturbations. We anticipate further exploration and application of the computational method to predict cellular responses of novel genetic perturbations in personalized medicine and genetic research.

## Supplementary Material

btae244_Supplementary_Data

## Data Availability

The data underlying this article are available in Gene Expression Omnibus at https://www.ncbi.nlm.nih.gov/geo/, and can be accessed with accession numbers GSE133344 (Norman et al. 2019) and GSE146194 (Replogle et al. 2022).
