# Supplementary material for: AttentionPert: accurately modeling multiplexed genetic perturbations with multi-scale effects
Source: Bioinformatics. 2024 Jun 28;40(Suppl 1):i453–61. doi: 10.1093/bioinformatics/btae244 (PMC11211811; doi:10.1093/bioinformatics/btae244)
Supplement: btae244_Supplementary_Data [file btae244_supplementary_data.pdf]

Supplementary Material for "AttentionPert: Accurately Modeling Multiplexed Genetic Perturbations with Multi-scale Effects"

Ding Bai, Caleb N. Ellington, Shentong Mo, Le Song*, Eric P. Xing*

March 27, 2024

Contents

1 Method Details **2**
1.1 Data preprocessing and loss functions . . . 2
1.2 An intuitive geometric illustration of our method . . . 2
1.3 Graphs and GNNs . . . 3
1.4 Non-additive features . . . 4

2 Evaluation Metric Details **4**

3 Hyperparameter Search **5**

4 Datasets details **6**

5 Additional Experiment Results **6**
5.1 Detailed results for comparison on all 5 splits of 3 datasets . . . 6
5.2 Detailed results of the ablation study . . . 7
5.2.1 Tesing on other 4 splits . . . 7
5.2.2 Testing random graphs with GO graph . . . 8
5.2.3 Testing a simple regression model . . . 8
5.3 Evaluations on different numbers of DE genes . . . 8

6 Perturbation-gene residual errors analysis **8**

*Corresponding authors. Le.Song@mbzuai.ac.ae and Eric.Xing@mbzuai.ac.ae

1 Method Details

1.1 Data preprocessing and loss functions

We use the three perturbational effects datasets [5, 6] preprocessed by GEARS [7] to evaluate our model. The preprocess of GEARS first normalizes the expression values, discard low-varying genes, then samples an unperturbed gene expression $\mathbf{x}_c^i$ from the set $\{\mathbf{x}^t, t = 1, ..., n_{\text{control}}\}$ to be 1-to-1 correspondent with each post-perturbation expression vector $\mathbf{y}_c^i$ where $i = 1, ..., n_c$. For the Gene Ontology (GO), we use the GO datasets provided by the SOTA method and also the Gene Ontology knowledgebase [1, 9, 8]. After aligning the genes, we have successfully found more GO terms for those genes not shown in the GO graph provided by GEARS.

We also utilize Gene2Vec [2] which are 200-dimension vector representations for human genes. We use a Gaussian distribution to sample the vector for genes shown in each of the datasets that cannot be aligned with the gene symbols provided by Gene2Vec. Formally, assuming the gene vector is $\mathbf{v}_g$ where $g \in \mathcal{G}$ is the set of gene symbols of Gene2Vec, for any unknown gene $x \notin \mathcal{G}$, its representation is sampled as:

$$\begin{cases} V_x \sim \mathcal{N}(\mu, \mathbf{\Sigma}) \\ \mu = \mathrm{E}\big[V_g\big] = \frac{1}{|\mathcal{G}|} \sum_{g \in \mathcal{G}} \mathbf{v}_g \\ \mathbf{\Sigma} = \mathrm{Cov}\big(V_g\big) = \frac{1}{|\mathcal{G}|} \sum_{g \in \mathcal{G}} (\mathbf{v}_g - \mu)(\mathbf{v}_g - \mu)^T \end{cases} \tag{1}$$

We follow the loss functions used by GEARS [7]. During the training, GEARS uses a loss function that measures the distance between each predicted expression $\hat{\mathbf{y}}_\theta(c)^i = \mathbf{g}_\theta(c) + \mathbf{x}_c^i$ and the ground-truth $\mathbf{y}_c^i$, which is:

$$\mathcal{L}^{\text{autofocus}}(\mathbf{y}_c^i) = \frac{1}{|Z_c|} \sum_{k \in Z_c} \big(\hat{\mathbf{y}}_\theta(c)_k^i - \mathbf{y}_{c,k}^i\big)^{2+\gamma} \tag{2}$$

$$\mathcal{L}^{\text{autofocus}}(\mathcal{B}) = \frac{1}{|\mathcal{B}|} \sum_{\mathbf{y}_c^i \in \mathcal{B}} \mathcal{L}^{\text{autofocus}}(\mathbf{y}_c^i) \tag{3}$$

, where $\mathcal{B}$ is a batch of post-perturbation expressions in the training process, Z_c is the set of genes with non-zero expressions in at least 1 cell under the perturbation condition c, and γ is a hyper-parameter which $= 2$ in GEARS experiment setup.

GEARS also combines a direction-aware loss with a parameter $\lambda = 0.1$ with the autofocus loss:

$$\mathcal{L}^{\text{direction}}(\mathbf{y}_c^i) = \frac{1}{|Z_c|} \sum_{k \in Z_c} \Big[\mathrm{sign}\big(\hat{\mathbf{y}}_\theta(c)_k^i - \mathbf{x}_{c,k}^i\big) - \mathrm{sign}\big(\mathbf{y}_{c,k}^i - \mathbf{x}_{c,k}^i\big)\Big]^2 \tag{4}$$

$$\mathcal{L}^{\text{direction}}(\mathcal{B}) = \frac{1}{|\mathcal{B}|} \sum_{\mathbf{y}_c^i \in \mathcal{B}} \mathcal{L}^{\text{direction}}(\mathbf{y}_c^i) \tag{5}$$

$$\mathcal{L}^{\text{train}}(\mathcal{B}) = \mathcal{L}^{\text{autofocus}}(\mathcal{B}) + \lambda \mathcal{L}^{\text{direction}}(\mathcal{B}) \tag{6}$$

.

In addition, the log variance in GEARS is taught to act as a proxy for model uncertainty by promoting its increase when errors are significant. This is achieved through a gene-specific layer that predicts the log variance for each gene, which is incorporated into the learning process via a modified Bayesian neural network loss, as detailed in [3]. To estimate the post-perturbation gene expression values, a Gaussian likelihood approach is employed. The uncertainty score serves as an indicator of the confidence level of the model when predicting outcomes for new perturbations.

1.2 An intuitive geometric illustration of our method

AttentionPert operates on the fundamental assumption that the unperturbed state of each gene can be represented as a vector within a D-dimensional hidden space. When certain genes undergo perturbation, we map these perturbation conditions to this high-dimensional space. This mapping alters the basal states of the genes into perturbed states, which are then decoded into scalar values representing the changes in post-perturbation expressions.

AttentionPert comprises two principal encoders: PertLocal and PertWeight. PertLocal is responsible for locally perturbing the basal states of genes. In contrast, PertWeight is designed to calculate both general and gene-specific effects on a more global scale. The terms *locality* and *globality* in this context are defined based on a gene-gene relationship graph. For our model, this graph is the Gene Ontology (GO) graph, which may be used in its standard form or in an augmented version. This entire sequence of events in the hidden space is illustrated in Fig 1.

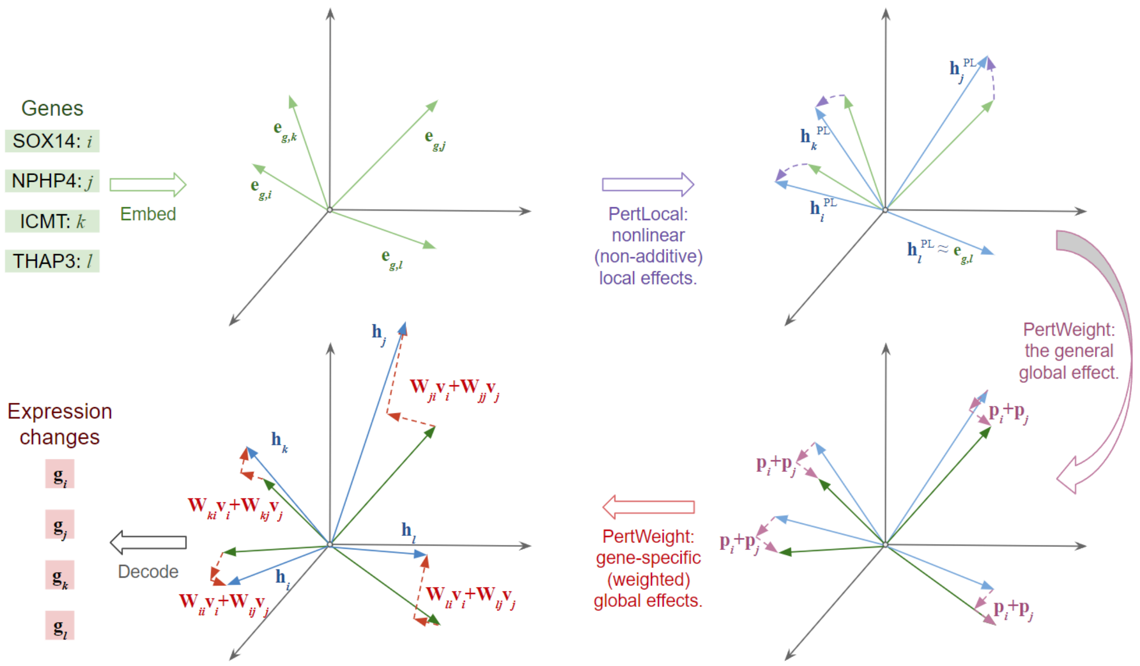

Figure 1: **The geometric illustration of AttentionPert.** First, genes i, j, k, l are embedded into a high-dimensional hidden space as vectors $\mathbf{e}_{g,i}, \mathbf{e}_{g,j}, \mathbf{e}_{g,k}, \mathbf{e}_{g,l}$. Assume that genes i and j are perturbed and are both local to k but distant from l in the GO graph. Then PertLocal locally (with non-additivity) perturbs each gene-representing vector into $\mathbf{H}^{\text{PL}}$. After that, PertWeight adds the general global effect $\mathbf{p}_i + \mathbf{p}_j$ to all vectors and finally adds a gene-specific global effect to each vector, resulting the encoding $\mathbf{H} = \mathbf{H}^{\text{PL}} + \mathbf{H}^{\text{PW}}$. The encoding for each gene is subsequently decoded into a scalar representing the expression change.

1.3 Graphs and GNNs

The GO graph is constructed by incorporating the shared GO terms of each pair gene [7], and those genes in the dataset that have no GO terms or have no intersected ones with other genes are still added to the graph as outlier nodes. Formally, for each pair of genes i and $j \in \{1, 2, ..., K\}$, denote the sets of corresponding Gene Ontology terms to be $\mathcal{N}_i$ and $\mathcal{N}_j$. Then the edge weight between these 2 genes is defined as $\mathbf{J}_{ij} = |\mathcal{N}_i \cap \mathcal{N}_j| / |\mathcal{N}_i \cup \mathcal{N}_j|$, which is the fraction of shared Gene Ontology terms between the 2 genes. After that, for each gene i, the $H_{\text{pert}} = 20$ gene j's with the highest $\mathbf{J}_{i,j}$ are selected to construct the Gene Ontology graph, and this selection gives each edge a direction since the top-weighted neighboring is not bidirectional. Hence the graph $\mathcal{J}$ is an edge-weighted directed graph that uses the Gene Ontology to construct gene-gene interactions, as shown in the main text Fig. 2.

Unlike GEARS, we also reduce the GO graph by not including possible perturbed genes that are not actually perturbed in the datasets. Instead of using all possible perturbed genes, we include all the genes in each of the three evaluation datasets to create the GO graph. In conclusion, the GO graph used by GEARS is the same graph of all possible perturbed genes for all three datasets, while our reduced GO graph is unique for each dataset that only contains all the genes from it. Fig. 4 shows that the GEARS using the reduced version of the GO graph usually makes better results than its original version.

We use SGConv [4] as the four GNN encoders in our model. A t-hop SGConv with a learnable weight matrix $\mathbf{T} \in \mathbb{R}^{D_{\text{in}} \times D_{\text{out}}}$ outputs:

$$\text{SGC}_{t,\mathbf{T}}(\mathbf{A}, \mathbf{E}) = \left(\hat{\mathbf{D}}_{\mathbf{A}}^{-1/2} \hat{\mathbf{A}} \hat{\mathbf{D}}_{\mathbf{A}}^{-1/2}\right)^t \mathbf{E}\mathbf{T} \tag{7}$$

, where $\hat{\mathbf{A}} = \mathbf{A} + \mathbf{I}$ denotes the adjacency matrix with inserted self-loops and $\hat{\mathbf{D}}_{\mathbf{A},ii} = \sum_{j=1}^{K} \hat{\mathbf{A}}_{ij}$ its diagonal degree matrix. The output is in $\mathbb{R}^{K \times D_{\text{out}}}$.

1.4 Non-additive features

As a perturbational effects prediction method, AttentionPert has theoretical non-additive features. Non-additivity requires the predicted $g_\theta(c)_k$ not always equal to $\sum_{i\in c} g_\theta(i)_k$ for any multi-gene perturbation c, and the non-linear coeffect of multiple perturbed genes can be learned.

The PertWeight encoder generates an additive encoding of any multi-gene condition c, formally,

$$\mathbf{H}^{\mathrm{PW}}(c) - \mathbf{H}^{\mathrm{PW}}(\emptyset) \equiv \sum_{i\in c} \Big(\mathbf{H}^{\mathrm{PW}}(\{i\}) - \mathbf{H}^{\mathrm{PW}}(\emptyset)\Big) \tag{8}$$

. Note that here $\mathbf{H}^{\mathrm{PW}}(\emptyset) \equiv 0$. However, PertLocal encoder utilizes the non-additive bias layer to avoid a linear combination of perturbed genes,

$$\mathbf{E}'_z = \mathbf{E}_z \big[1 + \beta(m-1)\tanh(\mathbf{K}_{\mathrm{NA}}\mathbf{z}_c)\big] \tag{9}$$

, where gene indices $i, j \in \{1, ..., K\}$. Since the function tanh is non-linear and m is the number of perturbed genes:

$$\mathbf{E}_z(\{i,j\}) \equiv \mathbf{E}_z(\{i\}) + \mathbf{E}_z(\{j\}) - \mathbf{E}_z(\emptyset) \tag{10}$$

$$\mathbf{E}'_z(\{i,j\}) \not\equiv \mathbf{E}'_z(\{i\}) + \mathbf{E}'_z(\{j\}) - \mathbf{E}'_z(\emptyset) \tag{11}$$

Therefore the PertLocal encoding is non-additive across all perturbed genes in any given condition c:

$$\mathbf{H}^{\mathrm{PL}}(c) - \mathbf{H}^{\mathrm{PL}}(\emptyset) \not\equiv \sum_{i\in c} \Big(\mathbf{H}^{\mathrm{PL}}(\{i\}) - \mathbf{H}^{\mathrm{PL}}(\emptyset)\Big) \tag{12}$$

. This implies that the final output is not additive for multi-gene perturbations either.

2 Evaluation Metric Details

The primary metric for evaluating the performance under each perturbation condition c is the Mean Squared Error (MSE). This metric measures the deviation between the expected prediction, approximated as the average predicted expressions $\mathrm{E}\big[Y_\theta(c)\big]$, and the actual ground-truth post-perturbation expression $\mathrm{E}\big[Y_c\big]$ focusing on the top 20 differentially expressed (DE) genes, denoted as $\mathcal{D}_c \subset 1, ..., K$ where $|\mathcal{D}_c| = 20$. The expected expression for both predicted and actual samples is calculated as the mean value across all cells under the given perturbation condition c. Formally:

$$\mathrm{E}\big[Y_c\big] \approx \bar{\mathbf{y}}_c = \frac{\sum_{i=1}^{n_c} \mathbf{y}_c^i}{n_c} \tag{13}$$

$$\mathrm{E}\big[Y_\theta(c)\big] \approx \bar{\mathbf{y}}_\theta(c) = \frac{\sum_{i=1}^{n_c} \mathbf{y}_\theta^i(c)}{n_c} \tag{14}$$

$$\mathrm{MSE(DE)}^\theta(c) = \frac{1}{20} \sum_{k\in\mathcal{D}_c} \Big(\mathrm{E}\big[Y_\theta(c)\big]_k - \mathrm{E}\big[Y_c\big]_k\Big)^2 \tag{15}$$

$$= \frac{1}{20} \sum_{k\in\mathcal{D}_c} \Big(\bar{\mathbf{y}}_\theta(c)_k - \bar{\mathbf{y}}_{c,k}\Big)^2 \tag{16}$$

Due to the substantial cost associated with CRISPR gene-perturbation screens, there is a pressing need for an in-silico method capable of predicting transcriptomic outcomes of novel single or multi-gene perturbations absent from existing datasets. In our experimental design, cells under a specific perturbation condition c are entirely allocated to either the training and validation set or the test set. This allocation strategy effectively simulates the challenge of predicting perturbations that the model has not previously encountered. For any given dataset, we define two distinct sets of perturbation conditions: $\mathcal{C}_{\mathrm{tv}}$, representing the training and validation set, and $\mathcal{C}_{\mathrm{test}}$, representing the test set. As all unperturbed cells are considered known and are therefore included in the training and validation set, the control condition is defined as $c_{\mathrm{control}} = \emptyset$ and is part of $\mathcal{C}_{\mathrm{tv}}$. This approach ensures a clear demarcation between training/validation and testing scenarios, crucial for evaluating the model's efficacy in handling novel perturbations.

When the number of perturbed genes is only 1, there is only one testing scenario that is called *seen 0/1*, because any perturbation condition containing only 1 gene which either shows in the train and validation set or shows in the test set. We denote such a scenario as $\mathcal{S}_{0/1} = \{c \in \mathcal{C}_{\mathrm{test}} || c| = 1\}$.

When considering the combination of multiple perturbed genes, however, there could be multiple testing scenarios, depending on the experimental exposure of the genes to perturbations during training. For s-gene perturbations test where $s > 1$ (in real-life datasets we only have $s = 2$), there could be $s + 1$ scenarios, which is $\mathcal{S}_{t/s} = \{c \in \mathcal{C}_{\text{test}} | |c| = s, |c \cap \bigcup_{c' \in \mathcal{C}_{\text{tv}}} c'| = t\}$ where $t = 0, 1, ...s$ meaning the number of genes in this perturbation condition that has shown in the train and validation sets. By that definition, over a s-perturbation test, a scenario with smaller t is a more OOD case and harder for the model to predict. The top 20 DE MSE metric of a test scenario $\mathcal{S}_{t/s}$ can be formulated as:

$$\text{MSE(DE)}^{\theta}(\mathcal{S}_{t/s}) = \frac{1}{|\mathcal{S}_{t/s}|} \sum_{c \in \mathcal{S}_{t/s}} \text{MSE}^{\theta}(c) \tag{17}$$

The relative MSE (rel-MSE) in percentage, which is the proportion relative to the Ctrl model, is calculated by:

$$\text{rel-MSE(DE)}^{\theta}(\mathcal{S}_{t/s}) = \frac{\text{MSE(DE)}^{\theta}(\mathcal{S}_{t/s})}{\text{MSE(DE)}^{\text{Ctrl}}(\mathcal{S}_{t/s})} \times 100\% \tag{18}$$

, and by definition of the Ctrl model that only outputs the average control sample:

$$\text{rel-MSE(DE)}^{\theta}(\mathcal{S}_{t/s}) = \frac{\sum_{c \in \mathcal{S}_{t/s}} \sum_{k \in \mathcal{D}_c} \left(\bar{\mathbf{y}}_{\theta}(c)_k - \bar{\mathbf{y}}_{c,k}\right)^2}{\sum_{c \in \mathcal{S}_{t/s}} \sum_{k \in \mathcal{D}_c} \left(\bar{\mathbf{x}}_k - \bar{\mathbf{y}}_{c,k}\right)^2} \times 100\% \tag{19}$$

In addition to MSE, we assess our model's performance using the Pearson correlation coefficient, which measures the relationship between the predicted and actual post-perturbation expression shifts. Formally:

$$\rho_{\Delta}(\text{all})^{\theta}(c) = \rho\Big(\text{E}\big[Y_{\theta}(c)\big] - \text{E}\big[X\big], \text{E}\big[Y_c\big] - \text{E}\big[X\big]\Big) \tag{20}$$

$$= \rho\Big(\bar{\mathbf{y}}_{\theta}(c) - \bar{\mathbf{x}}, \bar{\mathbf{y}}_c - \bar{\mathbf{x}}\Big) \tag{21}$$

, where $\rho(\cdot, \cdot)$ is the Pearson correlation coefficient of 2 vectors. The metric for a set of perturbations is also the average $\rho_{\Delta}(\text{all})^{\theta}$ over all perturbations in the given set. This coefficient is calculated across all genes to gauge the model's proficiency in predicting overall expression changes. MSE and Pearson scores can also be evaluated on different numbers of top DE genes, including 50, 100, and 200 in our implementation.

Beyond MSE and the Pearson score, we employ two additional metrics for a more comprehensive evaluation of predictive error rates of top 20 DE genes. The proportion of top 20 DE genes for a given perturbation predicted to change in an opposite direction of the ground truth is formally:

$$P_{\text{opposite direction}}(\text{DE})^{\theta}(c) = \frac{1}{20} \sum_{k \in \mathcal{D}_c} \mathbb{I}\Big(\text{sign}\big(\bar{\mathbf{y}}_{\theta}(c)^i_k - \bar{\mathbf{x}}^i_{c,k}\big) \neq \text{sign}\big(\bar{\mathbf{y}}^i_{c,k} - \bar{\mathbf{x}}^i_{c,k}\big)\Big) \tag{22}$$

. The proportion of DE genes for which the predicted post-perturbation gene expression falls beyond one standard deviation of the actual post-perturbation expression distribution mean is formally:

$$P_{\text{beyond one std}}(\text{DE})^{\theta}(c) = \frac{1}{20} \sum_{k \in \mathcal{D}_c} \mathbb{I}\Big(|\bar{\mathbf{y}}_{\theta}(c)^i_k - \bar{\mathbf{y}}^i_{c,k}| > \sigma_{c,k}\Big) \tag{23}$$

. Here $\mathbb{I}(\cdot)$ is the indicator function which equals 1 if the variable is true and 0 otherwise. The standard deviation of perturbation c on gene k: $\sigma_{c,k}$ is over the set of ground truth expressions : $\{\mathbf{y}^i_{c,k} | i = 1, ..., n_c\}$. Both metrics are averaged as the score of a set of perturbations as well.

3 Hyperparameter Search

We tune and fix the best set of hyperparameters on the validation set of the first split of the Norman dataset [5]. We search hyperparameters in ranges: the number of hops of SGConvs $t \in \{1, 2\}$; the network hidden size $D \in \{32, 64, 128\}$; for the multi-head attention, the number of heads $H \in \{1, 2, 4, 8\}$ and the dimension of each head $d_q \in \{8, 16, 32, 64, 128, 256\}$, where $H \times d_q \in$

Table 1: **Details of the perturbational effects datasets.** Here # means the number of something. The Norman dataset is a two-gene perturbation dataset [5]. The RPE1 and K562 datasets are single-gene perturbation datasets [6]. All three datasets are pre-processed by GEARS [7].

Dataset	# 1-gene perturbations	# 2-gene perturbations	# Cells	# 1-gene perturbed cells	# 2-gene perturbed cells	# Genes	Gene Ontology coverage (%)	Gene2Vec coverage (%)
Norman	105	131	91,205	48,407	35,445	5,045	63.2%	68.9%
RPE1	1,543	0	162,733	151,248	0	5,892	96.0%	97.8%
K562	1,092	0	162,751	152,060	0	5,680	94.6%	96.4%

Table 2: **Different splits of Norman, RPE1 and K562 datasets.** Here # means the number of something. For each dataset, the gene ratio of the train-validation set is 75%. The Norman dataset is a two-gene perturbation dataset [5] that contains 3 scenarios in the test set. The RPE1 and K562 datasets are single-gene perturbation datasets [6] with only seen 0/1 scenario. GEARS pre-processes all three datasets [7].

Dataset	Split	# Perturbed genes	# Perturbed genes in the train-val set	# Seen 0/1 perturbations	# Seen 0/2 perturbations	# Seen 1/2 perturbations	# Seen 2/2 perturbations
	1				9	52	18
	2				7	52	18
Norman	3	105	78	27	2	39	23
	4				6	44	21
	5				10	58	16
RPE1	all 5	1,543	1,157	386	0	0	0
K562	all 5	1,092	819	273	0	0	0

$[64, 256]$; the minimum edge weight parameter in the augmented GO graph $\alpha \in \{0, 0.25, 0.5, 0.75\}$; the NA-bias parameter $\beta \in \{0.001, 0.005, 0.01, 0.05\}$; batch size in $\{32, 64, 128, 256, 512\}$. The best hyperparameters are listed in the main body, where $t = 1, D = 64, H = 2, d_q = 64, \alpha = 0.75, \beta = 0.05$. To ensure a fair comparison with the GEARS model, we optimized the batch size, selecting 128 as the most effective value for both models. Additionally, we search the number of top-weighted edges retained in the Gene Ontology (GO) graph. The results indicated similar performance across a range of values $10, 15, 20, 25$, leading us to select 20, aligning with the configuration used in GEARS. Our model is versatile in its representation of prior knowledge regarding gene-gene relationships. While it can utilize various types of networks, such as protein-protein interactions or gene coessentiality networks, we choose to use the GO graph, as detailed in Supplementary Note 2 of GEARS [7]. This decision is driven by the desire to maximize gene coverage and maintain consistency for a fair comparison with GEARS.

4 Datasets details

Table 1 shows the details of each dataset, including the numbers of different perturbations, corresponding cell samples, and genes in each dataset covered in the Gene Ontology database or Gene2Vec. During the experiments, each dataset has 5 different splits for training, validation, and testing. We use the same split ratio of genes that the SOTA method GEARS conducts its experiments, where for both single-gene and multi-gene cases, the number of perturbed genes in the train and validation set is 75% of the total number of perturbed genes of the dataset. This makes 5 different sets of numbers of 3 scenarios in the 2-gene perturbational dataset [5] but the same number of the only 1 scenario for 1-gene datasets [6], shown in Table 2.

5 Additional Experiment Results

5.1 Detailed results for comparison on all 5 splits of 3 datasets

The difficulty across different splits of the same dataset can vary considerably since we do not randomly split each dataset across all cells. Instead, we ensure that all perturbations in the test set are not encountered during the training phase, leading to significant variability in model performance across different experimental splits. Therefore, rather than averaging evaluation metrics across all splits, we present a split-by-split comparison of the three datasets. Table 3 shows the

Table 3: **Seen 0/2, seen 1/2, and seen 2/2 comparison of MSE, relative MSE (%) and ρ_Δ on other 4 splits of Norman dataset.** MSE stands for the MSE of the top 20 DE genes. The relative mean squared error (rel-MSE) is in percentage (%). The ρ_Δ score refers to the Pearson score between the predicted shift and the ground-truth shift on all genes. We record the mean and the standard deviation over 5 independent experiments. The best performances of each split are marked in bold.

	Model	Seen 0/2			Seen 1/2			Seen 2/2		
		MSE(DE)	rel-MSE(DE)	ρ_Δ(all)	MSE(DE)	rel-MSE(DE)	ρ_Δ(all)	MSE(DE)	rel-MSE(DE)	ρ_Δ(all)
Split 2	Ctrl	0.798	100	−0.020	0.689	100	0.007	0.575	100	0.007
	CPA	0.546 ±0.004	68.4 ±0.5	0.576 ±0.009	0.380 ±0.008	55.1 ±1.2	0.584 ±0.011	0.191 ±0.020	33.1 ±3.5	0.637 ±0.005
	GEARS	0.403 ±0.032	50.4 ±4.0	0.591 ±0.023	0.241 ±0.011	35.0 ±1.7	0.621 ±0.010	0.101 ±0.004	17.6 ±0.6	0.643 ±0.014
	Ours	**0.329** ±0.021	**41.2** ±2.6	**0.592** ±0.011	**0.220** ±0.010	**31.9** ±1.5	**0.623** ±0.015	**0.088** ±0.007	**15.4** ±1.2	**0.679** ±0.007
Split 3	Ctrl	0.951	100	0.010	0.656	100	0.003	0.762	100	0.012
	CPA	0.542 ±0.067	57.0 ±7.1	0.569 ±0.070	0.300 ±0.031	45.8 ±4.7	**0.590** ±0.011	0.287 ±0.018	37.6 ±2.4	0.639 ±0.017
	GEARS	0.532 ±0.076	56.0 ±8.0	**0.593** ±0.047	0.208 ±0.018	31.7 ±2.7	0.585 ±0.016	0.146 ±0.009	19.2 ±1.1	0.651 ±0.018
	Ours	**0.532** ±0.100	**55.9** ±10.5	0.532 ±0.058	**0.198** ±0.014	**30.1** ±2.2	0.584 ±0.021	**0.137** ±0.012	**18.0** ±1.5	**0.651** ±0.024
Split 4	Ctrl	0.630	100	−5.609	0.607	100	0.008	0.727	100	0.007
	CPA	0.385 ±0.018	61.2 ±2.8	**0.538** ±0.014	0.285 ±0.000	47.0 ±0.1	**0.634** ±0.007	0.203 ±0.012	27.9 ±1.6	0.663 ±0.002
	GEARS	0.375 ±0.040	59.5 ±6.4	0.461 ±0.035	0.220 ±0.018	36.2 ±3.0	0.583 ±0.022	0.139 ±0.024	19.1 ±3.4	0.621 ±0.034
	Ours	**0.275** ±0.037	**43.7** ±5.9	0.516 ±0.016	**0.193** ±0.010	**31.8** ±1.7	0.622 ±0.004	**0.099** ±0.002	**13.6** ±0.3	**0.697** ±0.008
Split 5	Ctrl	0.637	100	0.006	0.670	100	0.013	0.722	100	0.018
	CPA	0.449 ±0.006	70.4 ±1.0	0.374 ±0.003	0.407 ±0.013	60.8 ±1.9	0.470 ±0.028	0.389 ±0.047	53.9 ±6.5	0.507 ±0.047
	GEARS	0.371 ±0.007	58.2 ±1.1	0.449 ±0.025	0.321 ±0.011	47.9 ±1.7	0.530 ±0.042	0.235 ±0.019	32.5 ±2.6	0.581 ±0.050
	Ours	**0.338** ±0.010	**53.0** ±1.6	**0.484** ±0.010	**0.281** ±0.004	**41.8** ±0.5	**0.578** ±0.011	**0.205** ±0.009	**28.4** ±1.2	**0.623** ±0.004

Table 4: **Seen 0/1 MSE, relative MSE (%) and ρ_Δ results on other 4 splits of RPE1 and K562 datasets.** The best performances of each split are marked in bold.

	Model	Data: RPE1			Data: K562		
		MSE(DE)	rel-MSE(DE)	ρ_Δ(all)	MSE(DE)	rel-MSE(DE)	ρ_Δ(all)
Split 2	Ctrl	0.282	100	−0.017	0.235	100	−0.015
	CPA	0.272 ±0.001	96.3 ±0.2	0.162 ±0.006	0.228 ±0.002	97.1 ±0.7	0.088 ±0.023
	GEARS	0.150 ±0.003	53.1 ±1.1	**0.512** ±0.011	0.145 ±0.002	61.9 ±0.7	0.358 ±0.013
	Ours	**0.147** ±0.006	**52.0** ±2.2	0.501 ±0.030	**0.130** ±0.004	**55.3** ±1.6	**0.376** ±0.010
Split 3	Ctrl	0.302	100	−0.017	0.249	100	−0.014
	CPA	0.296 ±0.002	97.8 ±0.6	0.118 ±0.025	0.245 ±0.001	98.4 ±0.5	0.081 ±0.011
	GEARS	0.169 ±0.009	56.1 ±3.1	0.482 ±0.031	0.161 ±0.004	64.4 ±1.6	0.345 ±0.012
	Ours	**0.156** ±0.008	**51.7** ±2.7	**0.505** ±0.015	**0.143** ±0.002	**57.5** ±0.8	**0.367** ±0.014
Split 4	Ctrl	0.312	100	−0.016	0.219	100	−0.014
	CPA	0.305 ±0.000	97.6 ±0.1	0.112 ±0.001	0.213 ±0.001	97.5 ±0.6	0.074 ±0.009
	GEARS	0.163 ±0.008	52.2 ±2.7	0.517 ±0.012	0.135 ±0.002	61.6 ±0.9	0.345 ±0.009
	Ours	**0.152** ±0.003	**48.6** ±1.1	**0.527** ±0.016	**0.120** ±0.003	**54.7** ±1.5	**0.378** ±0.012
Split 5	Ctrl	0.289	100	−0.016	0.225	100	−0.014
	CPA	0.279 ±0.002	96.6 ±0.5	0.141 ±0.019	0.221 ±0.001	98.0 ±0.4	0.082 ±0.018
	GEARS	0.153 ±0.006	53.1 ±2.2	0.506 ±0.012	0.143 ±0.002	63.4 ±0.9	0.345 ±0.008
	Ours	**0.148** ±0.005	**51.3** ±1.7	**0.509** ±0.016	**0.134** ±0.006	**59.5** ±2.5	**0.364** ±0.016

detailed MSE and ρ_Δ results on the other 4 splits of the Norman dataset (results for the first split are shown in the main body). Besides, percentages of DE genes with an opposite direction and beyond one standard deviation results of all 5 splits of the Norman dataset are shown in Fig 2. Results show that AttentionPert outperforms other methods in most of the metrics on every split when dealing with multi-gene perturbations.

The detailed MSE, rel-MSE and ρ_Δ results on the other 4 splits of RPE1 and K562 datasets (results for the first split are shown in the main body) are shown in Table 4, while error-rate representing results of all 5 splits of these two datasets are shown in Fig 3. Based on the experimental performances, AttentionPert also beats other methods in most of the metrics on every split for single-gene perturbation datasets.

5.2 Detailed results of the ablation study

5.2.1 Tesing on other 4 splits

The rel-MSE results for seen 0/2, seen 1/2 and seen 2/2 scenarios of the ablation experiments are shown in Fig. 4. We can see that AttentionPert does not beat all other revised GEARS models in seen 1/2 and seen 2/2 metrics. However, the results for seen 0/2 demonstrate that for the most OOD case where either of the perturbed genes is unseen when training, our model outperforms the revised version of GEARS and also any other methods. Therefore we still conclude that our

Table 5: **Comparison on Split 1 of Norman dataset for different graphs.** Here the model remains AttentionPert. *Random edges* means a graph of genes where each gene is assigned with 20 random edges and uniformly random weights in $[0, 1]$, and *Random weights* means a graph of genes where edges of the GO graph are not changed while edge weights are uniformly randomized in $[0, 1]$.

Graph	Seen 0/2		Seen 1/2		Seen 2/2	
	MSE(DE)	ρ_Δ(DE)	MSE(DE)	ρ_Δ(DE)	MSE(DE)	ρ_Δ(DE)
Random edges	0.209 ± 0.004	0.856 ± 0.005	0.225 ± 0.006	0.809 ± 0.002	**0.089** ± 0.010	**0.924** ± 0.004
Random weights	0.215 ± 0.032	0.845 ± 0.039	0.224 ± 0.003	0.799 ± 0.007	0.102 ± 0.000	0.906 ± 0.008
GO graph	**0.154** ± 0.007	**0.880** ± 0.007	**0.195** ± 0.010	**0.831** ± 0.010	0.097 ± 0.006	0.913 ± 0.004

Table 6: **Comparing the regression model with AttentionPert on unseen single gene perturbations on Split 1 of 2 datasets.**

Model	RPE1	K562
	MSE(DE)	MSE(DE)
Simple regression	0.302 ± 0.000	0.358 ± 0.003
AttentionPert	**0.132** ± 0.005	**0.132** ± 0.003

model generally performs better than any revised version of GEARS.

5.2.2 Testing random graphs with GO graph

Our further experiment results in Table 5 show that the model with random graphs generated by shuffling the edges, cannot compete with the model with the true GO graph, especially in the seen 0/2 scenario. It is shown that the GO graph provides useful gene-gene interaction information, rather than simply working as a regularizer.

5.2.3 Testing a simple regression model

For the prediction of the unseen single gene perturbations, our model is also compared with simple regression models where the perturbational response matrix $\mathbf{Y} \in \mathbb{R}^{K \times K}$ is generated by the covariate matrix $\mathbf{X} \in \mathbb{R}^{K \times D_e}$ initialized by the Gene2Vec embeddings (columns) for each perturbed gene (rows). The results in Table 6 show that AttentionPert outperforms a simple regression model largely.

5.3 Evaluations on different numbers of DE genes

Metrics including MSE and Pearson correlation scores for the top 20, 50, 100 and 200 differentially expressed (DE) genes are all evaluated, with results shown in Table 7. The performances demonstrate that AttentionPert consistently outperforms baselines on metrics for different numbers of top DE genes.

6 Perturbation-gene residual errors analysis

While AttentionPert is state-of-the-art in terms of performance and generalization, it is far from a perfect model of the underlying biological system. We present an error analysis in Fig. 5 of the main text to highlight this and triage opportunities for future research based on apparent machine learning and biological insights.

In supplementary, we also plot MSEs with column-scaling by test groups (Fig. 5), with no scaling (Fig. 6), with row-scaling (Fig. 7), as well as by mean and variance of post-perturbation expression changes (Fig. 8). Little correlation with errors by test groups can be found. We do see small correlations with the magnitude and standard deviation of post-perturbation expression changes. Fig. 9 also shows that the error differences cannot be explained by graph connectivity. The best directions for future developments and shortcomings of AttentionPert seem to be the biological indications seen in Fig. 6, with large perturbation effects being the most difficult to predict (Fig. 8).

Table 7: **Comparison on Split 1 of Norman dataset for different numbers of DE.** Here #DE means the number of top differentially expressed genes.

#DE	Model	Seen 0/2		Seen 1/2		Seen 2/2	
		MSE(DE)	ρ_Δ(DE)	MSE(DE)	ρ_Δ(DE)	MSE(DE)	ρ_Δ(DE)
20	CPA	0.243 $\pm$0.032	0.851 $\pm$0.023	0.388 $\pm$0.010	0.744 $\pm$0.017	0.321 $\pm$0.026	0.740 $\pm$0.026
	GEARS	0.219 $\pm$0.011	0.862 $\pm$0.010	0.221 $\pm$0.007	0.819 $\pm$0.012	0.130 $\pm$0.007	0.855 $\pm$0.015
	Ours	**0.154** $\pm$0.007	**0.880** $\pm$0.007	**0.195** $\pm$0.010	**0.831** $\pm$0.010	**0.097** $\pm$0.006	**0.913** $\pm$0.004
50	CPA	0.115 $\pm$0.014	0.724 $\pm$0.019	0.187 $\pm$0.002	0.686 $\pm$0.010	0.167 $\pm$0.009	0.729 $\pm$0.012
	GEARS	0.111 $\pm$0.004	0.722 $\pm$0.014	0.109 $\pm$0.003	0.772 $\pm$0.008	0.078 $\pm$0.004	0.851 $\pm$0.017
	Ours	**0.087** $\pm$0.003	**0.760** $\pm$0.008	**0.096** $\pm$0.004	**0.785** $\pm$0.011	**0.062** $\pm$0.003	**0.898** $\pm$0.004
100	CPA	0.191 $\pm$0.002	0.748 $\pm$0.003	0.139 $\pm$0.004	0.676 $\pm$0.006	0.075 $\pm$0.004	0.775 $\pm$0.003
	GEARS	0.136 $\pm$0.010	0.779 $\pm$0.029	0.090 $\pm$0.004	0.747 $\pm$0.010	0.047 $\pm$0.002	0.782 $\pm$0.009
	Ours	**0.113** $\pm$0.004	**0.788** $\pm$0.010	**0.086** $\pm$0.002	**0.772** $\pm$0.010	**0.043** $\pm$0.003	**0.804** $\pm$0.015
200	CPA	0.056 $\pm$0.005	0.670 $\pm$0.031	0.086 $\pm$0.001	0.701 $\pm$0.006	0.061 $\pm$0.002	0.696 $\pm$0.010
	GEARS	0.053 $\pm$0.001	0.679 $\pm$0.012	0.053 $\pm$0.001	0.776 $\pm$0.010	0.038 $\pm$0.003	0.817 $\pm$0.019
	Ours	**0.046** $\pm$0.001	**0.701** $\pm$0.008	**0.051** $\pm$0.000	**0.787** $\pm$0.007	**0.034** $\pm$0.001	**0.849** $\pm$0.005

While this is not the case for the majority of predictions, in some cases the residual errors are very high, indicating poor generalization on select scenarios. In the highest error perturbations shown in Table 8, we see SNAI1 and ZBTB10 frequently shown in difficult perturbation conditions, and the expression change of HBZ is the hardest to predict. Surprisingly, many of the highest errors are Haemoglobin complex subunits. This again reinforces our notion from Fig. 6 that there are nuances in post-perturbation transcriptome dynamics that are not learned by AttentionPert.

References

[1] Seth Carbon, Amelia Ireland, Christopher J Mungall, ShengQiang Shu, Brad Marshall, Suzanna Lewis, AmiGO Hub, and Web Presence Working Group. Amigo: online access to ontology and annotation data. *Bioinformatics*, 25(2):288–289, 2009.

[2] Jingcheng Du, Peilin Jia, Yulin Dai, Cui Tao, Zhongming Zhao, and Degui Zhi. Gene2vec: distributed representation of genes based on co-expression. *BMC genomics*, 20:7–15, 2019.

[3] Alex Kendall and Yarin Gal. What uncertainties do we need in bayesian deep learning for computer vision? *Advances in neural information processing systems*, 30, 2017.

[4] Yuhong Li, Tianle Cai, Yi Zhang, Deming Chen, and Debadeepta Dey. What makes convolutional models great on long sequence modeling? *arXiv preprint arXiv:2210.09298*, 2022.

[5] Thomas M Norman, Max A Horlbeck, Joseph M Replogle, Alex Y Ge, Albert Xu, Marco Jost, Luke A Gilbert, and Jonathan S Weissman. Exploring genetic interaction manifolds constructed from rich single-cell phenotypes. *Science*, 365(6455):786–793, 2019.

[6] Joseph M Replogle, Reuben A Saunders, Angela N Pogson, Jeffrey A Hussmann, Alexander Lenail, Alina Guna, Lauren Mascibroda, Eric J Wagner, Karen Adelman, Gila Lithwick-Yanai, et al. Mapping information-rich genotype-phenotype landscapes with genome-scale perturb-seq. *Cell*, 185(14):2559–2575, 2022.

[7] Yusuf Roohani, Kexin Huang, and Jure Leskovec. Predicting transcriptional outcomes of novel multigene perturbations with gears. *Nature Biotechnology*, pages 1–9, 2023.

[8] Paul D Thomas, Dustin Ebert, Anushya Muruganujan, Tremayne Mushayahama, Laurent-Philippe Albou, and Huaiyu Mi. Panther: Making genome-scale phylogenetics accessible to all. *Protein Science*, 31(1):8–22, 2022.

[9] Paul D Thomas, David P Hill, Huaiyu Mi, David Osumi-Sutherland, Kimberly Van Auken, Seth Carbon, James P Balhoff, Laurent-Philippe Albou, Benjamin Good, Pascale Gaudet, et al. Gene ontology causal activity modeling (go-cam) moves beyond go annotations to structured descriptions of biological functions and systems. *Nature genetics*, 51(10):1429–1433, 2019.

Table 8: **The 20 tested perturbation-gene pairs with the highest residual errors of Split 1 for the Norman dataset.** Here the true expression changes and the std values are also shown. These are among 79 tested perturbations and 304 genes in the union set of top 20 DE genes of these conditions.

Perturbation	Gene	MSE	True Expression Change	True Expression Change Std
SNAI1+ZBTB10	HBZ	5.858342	0.882295	0.815754
SNAI1+ZBTB10	HBG2	3.797963	1.009285	0.931898
COL2A1+KLF1	COL2A1	3.525544	1.786843	0.883451
ELMSAN1+ZBTB10	HBZ	3.432350	0.578010	1.072782
DLX2+ZBTB10	HBZ	3.166230	1.006082	0.830266
CBL+PTPN12	HBZ	2.963096	2.854542	1.138285
SNAI1+ZBTB10	CTSC	2.511957	1.464524	1.425349
CBL+CNN1	SLC25A37	2.464964	2.032383	1.108583
DUSP9+KLF1	HBZ	2.204732	3.031719	0.844125
PTPN12+ZBTB10	HBZ	2.201152	0.059879	1.321451
DLX2+ZBTB10	HBG2	2.168637	1.022457	0.879665
DUSP9+ETS2	HBZ	2.106529	1.747679	1.127463
DUSP9+SNAI1	DUSP9	2.102543	1.365422	0.876005
DUSP9+SNAI1	HBZ	2.044535	0.216298	1.313410
CBL+CNN1	HBZ	2.025131	3.092346	1.295008
CNN1+UBASH3A	SLC25A37	2.015901	1.971746	1.344287
COL2A1+KLF1	HBZ	1.903269	0.215524	1.216313
DUSP9+IGDCC3	DUSP9	1.859017	1.298249	0.796284
DLX2+ZBTB10	FCER1G	1.855931	1.402502	1.046106
DUSP9+KLF1	DUSP9	1.850590	1.316458	0.646431

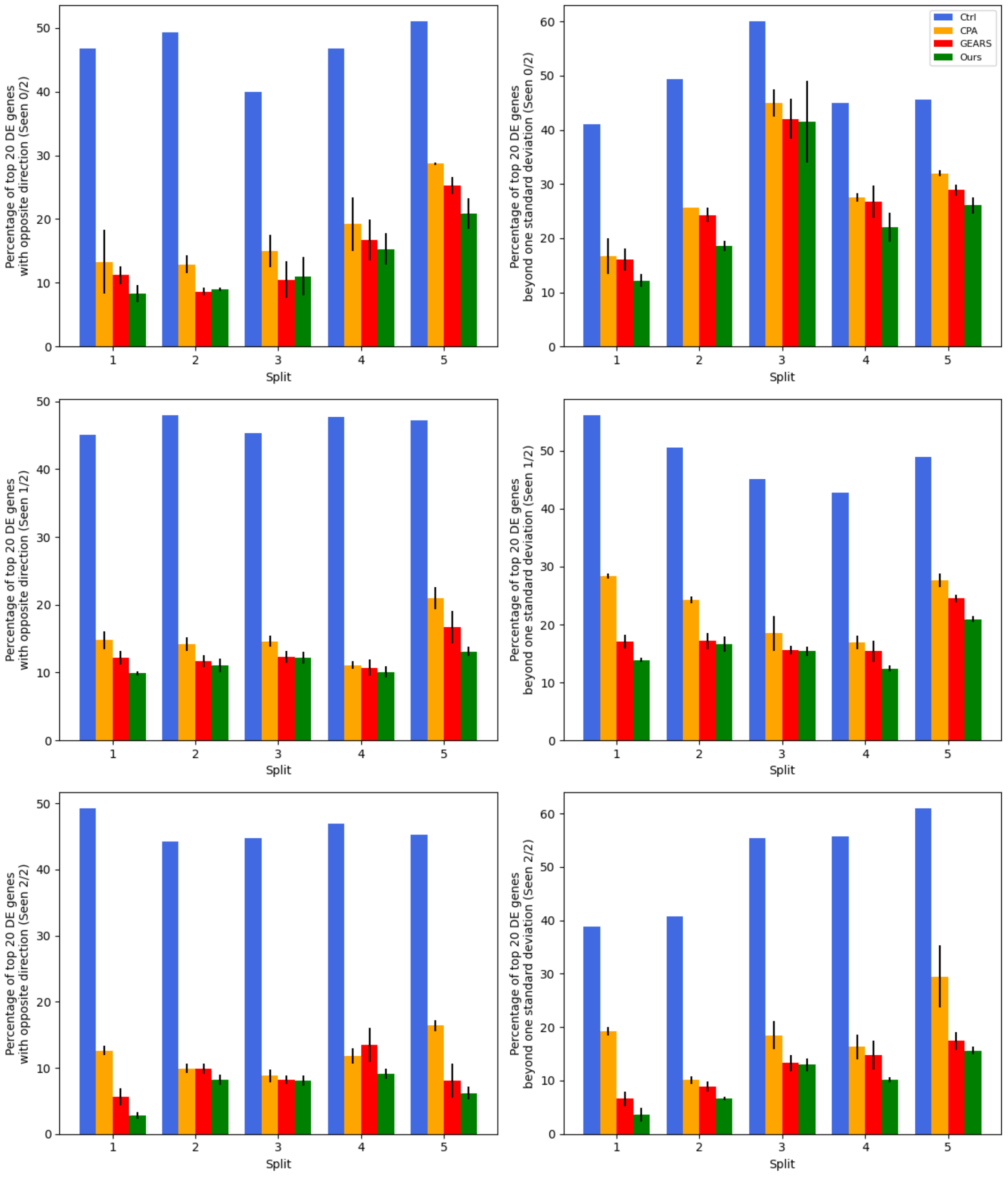

Figure 2: **Comparison of two error-rate representing metrics on Norman dataset.**

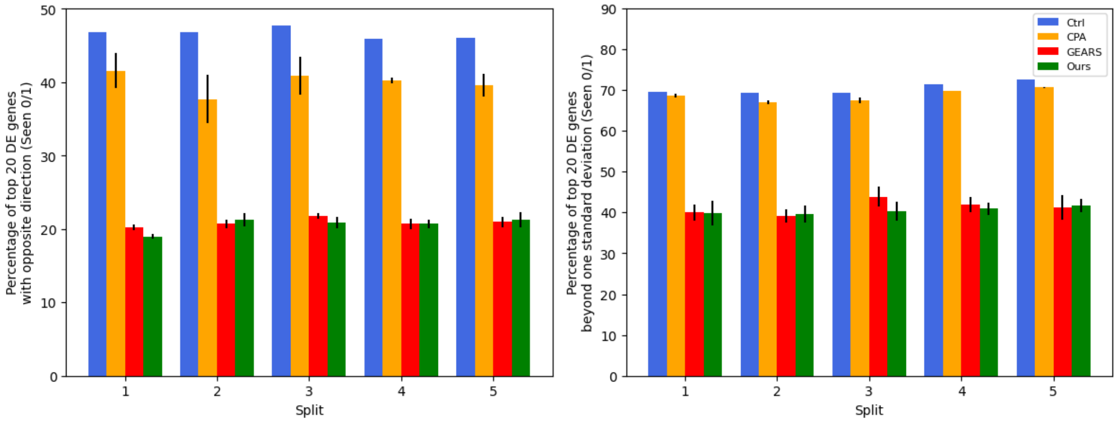

(a) RPE1

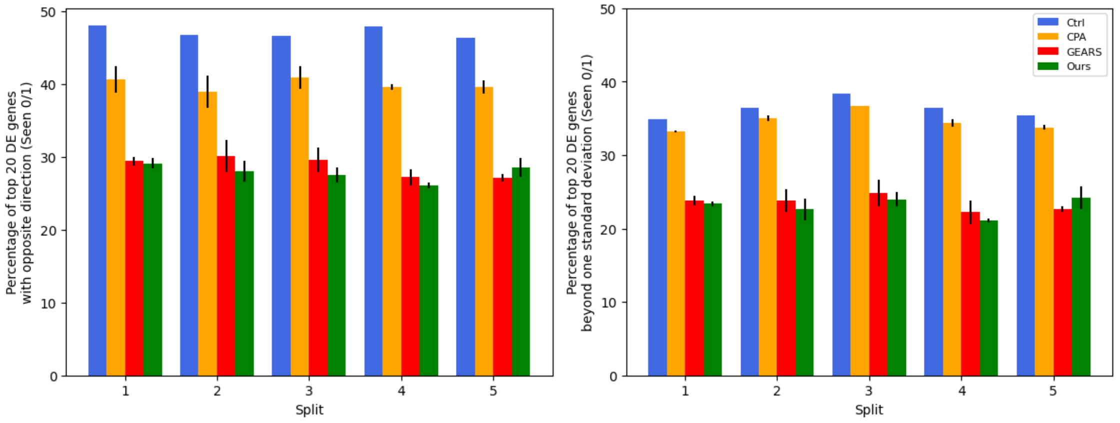

(b) K562

Figure 3: **Comparison of two error-rate representing metrics of RPE1 (a) and K562 (b) datasets.** Both are single-gene perturbation datasets with only seen 0/1 to compare.

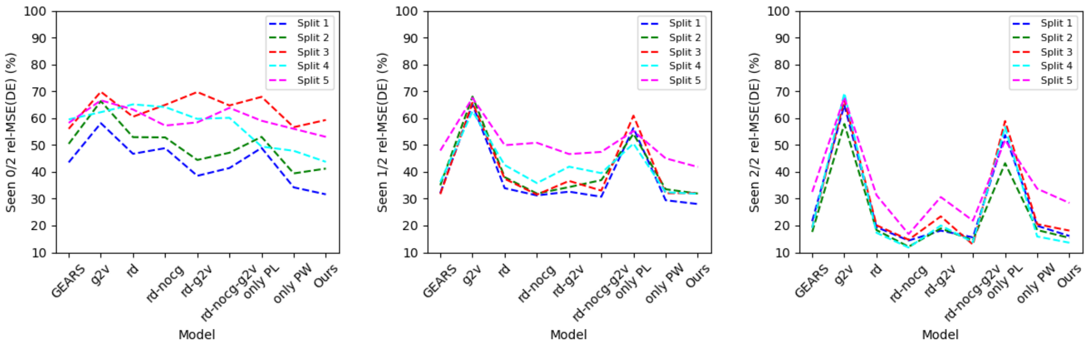

(a) Seen 0/2 (b) Seen 1/2 (c) Seen 2/2

Figure 4: **The ablation study comparing rel-MSE (%) of top 20 DE genes over seen 0/2 (a), seen 1/2 (b) and seen 2/2 scenarios (b), on 5 splits of Norman dataset**. Here, *g2v* is GEARS that also uses Gene2Vec to initialize the gene embedding, *rd* model is GEARS changed by merely reducing the possible perturbed genes to the genes in the dataset, and *nocg* is GEARS without the cross-gene layer. For our design, *only PL/PW* means the model with only the PertLocal/PertWeight encoder.

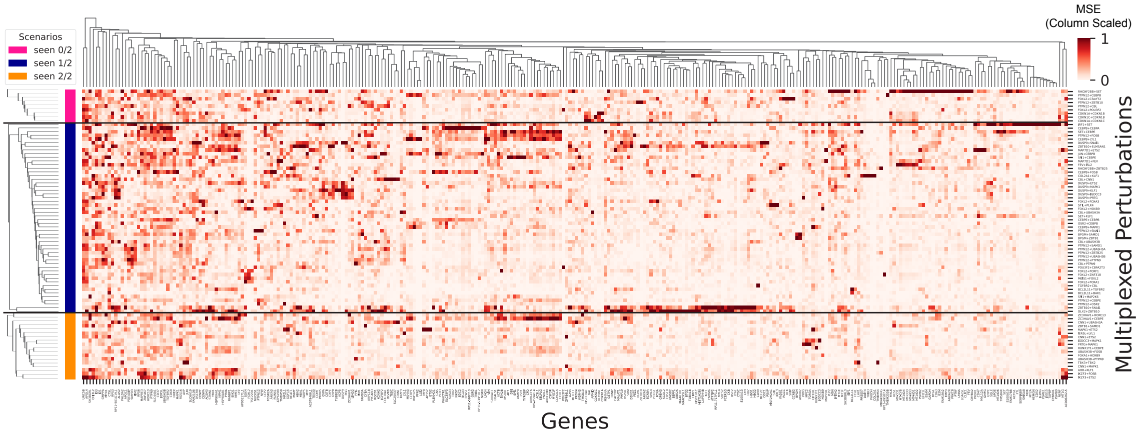

Figure 5: **Column scaled MSEs of AttentionPert, grouped by perturbation condition set.** The results are shown over 79 tested 2-gene perturbations of Split 1 in the Norman dataset as rows and the union set of the top 20 DE genes of these perturbations as columns.

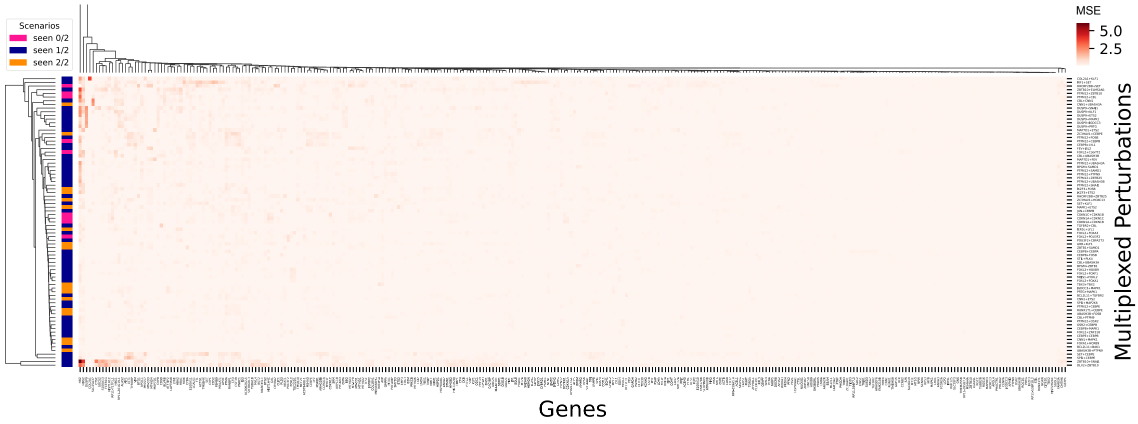

Figure 6: **MSEs of AttentionPert by gene and perturbation condition, no scaling.**

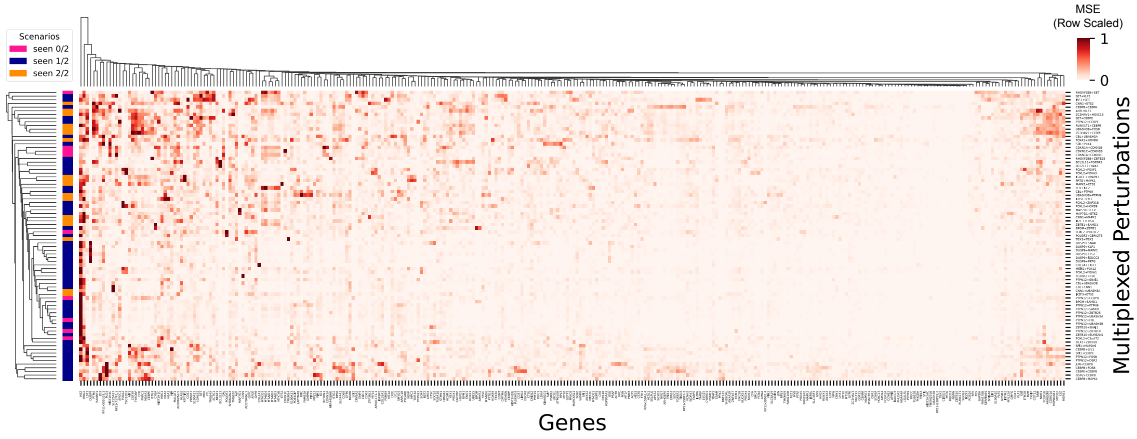

Figure 7: **Row-scaled MSEs of AttentionPert by gene and perturbation condition.**

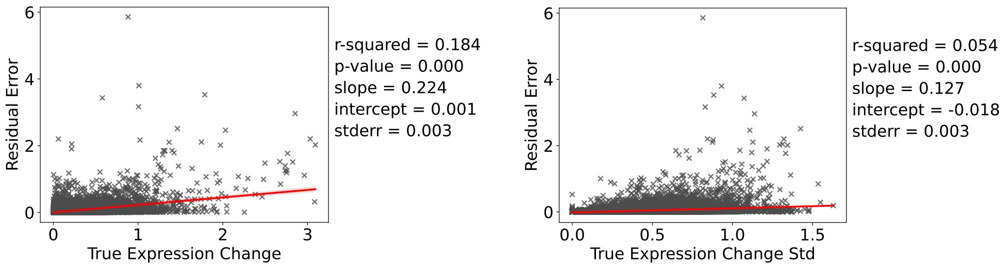

(a) Mean expression change absolute value vs. residual error

(b) Mean expression change standard deviation vs. residual error.

Figure 8: True post-perturbation expression changes mean (a) and standard deviation (b) vs AttentionPert MSEs.

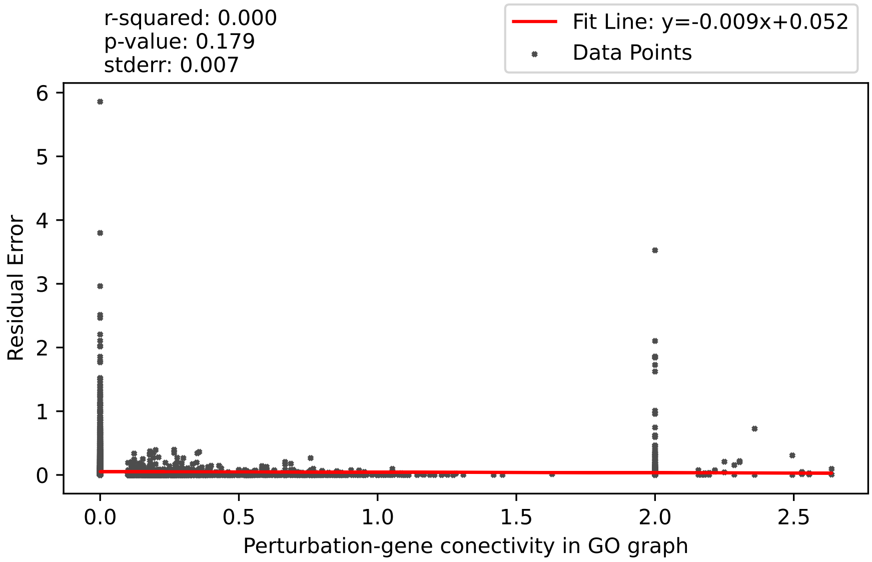

Figure 9: **Connectivity of perturbation-gene pair vs. AttentionPert MSE.** The connectivity is defined as the sum of edge weights between the perturbed genes and the affected gene in the Gene Ontology graph.
